# Human CD34^+^-derived plasmacytoid dendritic cells as surrogates for primary pDCs and potential cancer immunotherapy

**DOI:** 10.3389/fimmu.2024.1433119

**Published:** 2024-11-07

**Authors:** Giovanna Fiore, Wolfgang Weckwarth, Kerstin Paetzold, Llucia Albertí Servera, Manuela Gies, Jakob Rosenhauer, Martina Antoniolli, Sina Nassiri, Stephan Schmeing, Steffen Dettling, Bhavesh Soni, Meher Majety, Anne B. Krug, Sabine Hoves, Monika Julia Wolf

**Affiliations:** ^1^ Roche Pharma Research and Early Development (pRED), Roche Innovation Center Munich, Penzberg, Germany; ^2^ Roche Pharma Research and Early Development (pRED), Roche Innovation Center Basel, Basel, Switzerland; ^3^ Roche Pharma Research and Early Development (pRED), Roche Innovation Center Zurich, Zurich, Switzerland; ^4^ Institute for Immunology, Biomedical Center (BMC), Faculty of Medicine, LMU Munich, Munich, Germany

**Keywords:** dendritic cell differentiation, hematopoietic stem cells (HSCs), human, DC vaccination, cancer immunotherapy, plasmacytoid dendritic cells (pDCs)

## Abstract

**Introduction:**

Plasmacytoid dendritic cells (pDCs) are capable of triggering broad immune responses, yet, their scarcity in blood coupled to their reduced functionality in cancer, makes their therapeutic use for *in situ* activation or vaccination challenging.

**Methods:**

We designed an *in vitro* differentiation protocol tailored for human pDCs from cord blood (CB) hematopoietic stem cells (HSCs) with StemRegenin 1 (SR-1) and GM-CSF supplementation. Next, we evaluated the identity and function of CB-pDCs compared to human primary pDCs. Furthermore, we tested the potential of CB-pDCs to support anti-tumor immune responses in co-culture with tumor explants from CRC patients.

**Results:**

Here, we report an *in vitro* differentiation protocol enabling the generation of 200 pDCs per HSC and highlight the role of GM-CSF and SR-1 in CB-pDC differentiation and function. CB-pDCs exhibited a robust resemblance to primary pDCs phenotypically and functionally. Transcriptomic analysis confirmed strong homology at both, baseline and upon TLR9 or TLR7 stimulation. Further, we could confirm the potential of CB-pDCs to promote inflammation in the tumor microenvironment by eliciting cytokines associated with NK and T cell recruitment and function upon TLR7 stimulation *ex vivo* in patient tumor explants.

**Discussion:**

This study highlights CB-pDCs as surrogates for primary pDCs to investigate their biology and for their potential use as cell therapy in cancer.

## 1 Introduction

Plasmacytoid dendritic cells (pDCs) are a rare innate immune cell type specialized in massive secretion of type I interferons (IFNs) (1). Upon sensing viral elements such as dsDNA and ssRNA via toll-like receptor 7 (TLR7) and TLR9 respectively, IRF7, and NFkB signaling pathways are activated leading to IFN-α and pro-inflammatory cytokine secretion besides up-regulation of co-stimulatory molecules by pDCs (2, 3). Multifaceted activation of pDCs was extensively described to support antiviral immune responses through activation of innate and adaptive effector cells including NK and T cells (4). Their critical role was reported in SARS-CoV-2 and HSV infections in humans and in murine MCMV and coronavirus models (5–8).

Apart from their pro-inflammatory function, pDCs were shown to infiltrate several tumor types, adopting a tolerogenic phenotype (9–11). Across indications, tumor-associated pDCs (TA-pDCs) were characterized by the incapacity to produce type I IFN in response to TLR stimulation (10, 12, 13). Furthermore, TA-pDCs actively participate in establishing and promoting an immunosuppressive tumor microenvironment (TME). In melanoma, breast, and ovarian cancer, TA-pDCs promote regulatory T cell expansion via costimulation with ICOS-L (14–16). In non-small cell lung cancer, IL-1α, a pro-angiogenic and pro-invasive factor, was shown to be secreted by TA-pDCs (17).

Nonetheless, pDCs remain targets of choice for cancer immunotherapy due to their unique ability to release type I IFNs and to engage multiple players of immunity (18). Hence, several clinical trials evaluate the potential of TLR7 or TLR9 agonists in various tumor indications including head and neck cancer, melanoma, and esophageal cancer (NCT04799054, NCT00669292). Imiquimod, a synthetic TLR7 agonist approved for basal cell carcinoma, remains a successful illustration of *in situ* activation of TA-pDCs translating into tumor regression (19–22). Besides directly activating TA-pDCs, autologous pDC vaccination is also being tested for cancer therapy (23). The potential of pDC vaccination was investigated by Tel et al. in a phase I clinical trial enrolling melanoma patients (24). Vaccination with tumor-antigen pulsed pDCs elicited potent anti-tumor immune responses and resulted in increased overall survival compared to standard of care. Albeit promising, pDC vaccination shows major obstacles mainly due to the scarcity and fragility of pDCs, which limits the amount of cells to be used for vaccination (23, 25). To overcome these challenges, *ex vivo* manufacturing is an adequate strategy to generate clinically applicable numbers of pDCs. These protocols usually differentiate hematopoietic stem cells (HSCs) from cord blood (CB) or peripheral blood into DCs using combinations of growth factors (26). Recently, *in vitro* differentiated pDCs alone and in combination with other DC subtypes were shown to promote anti-tumor immune responses *in vivo* in leukemia models, highlighting their untapped potential for cancer immunotherapy (27, 28).

Although many studies reported *in vitro* differentiation of several DC subtypes, the yield of cells and functionality upon stimulus remains a challenge. Further, their resemblance to blood counterparts was often incompletely characterized (29–31). In the last few years, significant progress has been made to resolve these shortcomings. Balan et al. described an expansion step to significantly increase the numbers of HSCs before their differentiation into DCs (32). Another report underlined the importance of IFN-β and IFN-ɣ treatment (so-called IFN priming) to overcome unresponsiveness of *in vitro* differentiated pDCs towards a canonical stimulus (33). However, only a handful of protocols were dedicated to generate numbers of pDCs that could be relevant for therapeutic application (32, 34). Extensive characterization of *in vitro* differentiated pDCs in a head-to-head comparison with primary pDCs remains an essential prerequisite for their use in clinical settings.

Here, we demonstrate that CB-pDCs are adequate surrogates for primary pDCs. We describe a versatile *in vitro* differentiation protocol enabling the generation of human pDCs from HSCs in clinically relevant numbers. We demonstrate that CB-pDCs resemble *bona fide* pDCs phenotypically, transcriptionally and functionally. Importantly, we show the potential of CB-pDCs to inflame the TME *in vitro*, thus supporting their use for cell therapy in cancer.

## 2 Materials and methods

### 2.1 Materials

#### 2.1.1 Key resources table

**Table 1:** List of reagents and resources.

Reagent or Resource	Source	Identifier
Antibodies		
Anti-human CD1c PE/Cy7 (clone L1619)	Biolegend	Cat#: 331515; RRID: AB_1953227
Anti-human CD4 APC (clone OKT4)	Biolegend	Cat#:317415; RRID: AB_571944
Anti-human CD11c BUV737 (clone B-ly6)	BD Biosciences	Cat#:741827; RRID: AB_2871162
Anti-human CD40Pe/Cy7 (clone HB14)	Biolegend	Cat#:311011; RRID: AB_2563922
Anti-human CD45 BUV395 (clone HI30)	BD Biosciences	Cat#:563791; RRID: AB_2869519
Anti-human CD45 BUV805 (clone HI30)	BD Biosciences	Cat#:612891; RRID: AB_2870179
Anti-human CD45 PerCP (clone 2D1)	Biolegend	Cat#:304025; RRID: AB_893341
Anti-human CD45RA BV510 (clone HI100)	Biolegend	Cat#:304141; RRID: AB_2561384
Anti-human CD56 BV421 (clone HCD-56)	Biolegend	Cat#:318327; RRID: AB_10900228
Anti-human CD62L BV605 (clone DREG-56)	Biolegend	Cat#:304833; RRID: AB_2562129
Anti-human CD69 BUV737 (clone FN50)	BD Biosciences	Cat#:612818
Anti-human CD80 BUV395 (clone L307)	BD Biosciences	Cat#:565210; RRID: AB_2739112
Anti-human CD83 BV711 (clone HB15e)	BD Biosciences	Cat#:740802; RRID: AB_2740465
Anti-human CD86 BV421 (clone IT2.2)	Biolegend	Cat#:305425; RRID: AB_10899582
Anti-human CD86 BV605 (clone 2331)	BD Biosciences	Cat#:562999; RRID: AB_2737941
Anti-human CD123 BV650 (clone 6H6)	Biolegend	Cat#:306019; RRID: AB_11218792
Anti-human CD141 BV711 (clone 1A4)	BD Biosciences	Cat#:563155; RRID: AB_2738033
Anti-human CD197 (CCR7) PE (clone G043H7)	Biolegend	Cat#:353203; RRID: AB_10916391
Anti-human CD197 (CCR7) PerCPeFluor710 (clone 3D12)	ThermoFisher	Cat#:46-1979-42; RRID: AB_10853814
Anti-human CD252 (OX-40L) BV421 (clone ik-1)	BD Biosciences	Cat#:563766; RRID: AB_2738412
Anti-human CD253 (TRAIL) APC (clone RIK-2)	Biolegend	Cat#:308209; RRID: AB_2564397
Anti-human CD274 (PD-L1) BV605 (clone MIH1)	BD Biosciences	Cat#:563469
Anti-human CD303 (CLEC4C) APC (clone 201A)	Biolegend	Cat#:354205; RRID: AB_11147168
Anti-human CD304 BV510 (clone 12C2)	Biolegend	Cat#:354515; RRID: AB_2563074
Anti-human CD304 PerCP Cy5.5 (clone 12C2)	Biolegend	Cat#:354509; RRID: AB_2561558
Anti-human CD314 (NKG2D) BV711 (clone P30-15)	Biolegend	Cat#:320802; RRID: AB_492956
Anti-human CD327 (SIGLEC6) APC (clone 767329)	R&D Systems	Cat#:FAB2859A
Anti-human CD370 (CLEC9A) APC (clone M80)	Biolegend	Cat#:35805; RRID: AB_2565518
Anti-human AXL PE (clone 108724)	R&D Systems	Cat#:FAB154P
Anti-human HLA-ABC BUV661 (clone G46-2.6)	BD Biosciences	Cat#:741621; RRID: AB_2871027
Anti-human HLA-DR BV711 (clone L243)	BD Biosciences	Cat#:563696; RRID: AB_2738378
Anti-human IFN-α2 FITC (clone MMHA-1)	PBL Assay Science	Cat#:21112-3
Anti-human lineage (CD3, CD14, CD16, CD19, CD20, CD56) FITC (clones OKT3; M5E2; 3G8; HIB19; 2H7; HCD56)	Biolegend	Cat#:348801; RRID: AB_10612570
Human Fc Receptor Blocking Solution (TrueStain FcX)	Biolegend	Cat#422302
Biological samples		
Human peripheral blood mononuclear cells (PBMCs)	Roche Diagnostics Medical Service	N/A
Human cord blood CD34^+^ hematopoietic stem and progenitor cells (HSCs)	Lonza	Cat#:2C-101
Tumor digests from patients	Discovery Life Science	N/A
Tumor pieces from patients	Fidelis	N/A
Tumor pieces from patients	Indivumed	N/A
Chemicals, peptides, and recombinant proteins		
2-Mercaptoethanol	PAN Biotech	Cat#:P07-05020
Accutase	PAN Biotech	Cat#:P10-2110M
Albumin from bovine serum 30%	Sigma-Aldrich	Cat#:A9576
Collagenase IV	Worthington	Cat#:LS0004186
CpG-A ODN 2216 TLR9	Invivogen	Cat#:tlrl-2216
DNAse I, type 4	Sigma-Aldrich	Cat#:D5025
DPBS (1x)	PAN Biotech	Cat#:P04-36500
DMSO	Sigma-Aldrich	Cat#:D8418
heat-inactivated FBS	Anprotech	
hepes buffer	Sigma-Aldrich	Cat#:51558
human serum albumin	Sigma-Aldrich	Cat#:SRP6182
Hyaluronidase	Sigma-Aldrich	Cat#:H3506
L-ascorbic acid	Sigma-Aldrich	Cat#:A92902
L-glutamin	Anprotech	AC-AS-0001
MEM-α with nucleosides	GIBCO	Cat#:12571063
MEM non-essential amino acid	PAN Biotech	Cat#:P08-32100
MEM vitamin solution	PAN Biotech	Cat#:11120052
Mitomycin C	Sigma-Aldrich	Cat#:M4287
penicillin/streptomycin	Anprotech	Cat#:P06-07100
R848 Resiquimod	Invivogen	Cat#:tlrl-r848
Recombinant human FLT3-ligand	Peprotech	Cat#:300-19
Recombinant human GM-CSF (carrier-free)	Biolegend	Cat#:572905
Recombinant human IFN-β	Peprotech	Cat#:AF-300-02B
Recombinant human IFN-γ	Peprotech	Cat#:300-02BC
Recombinant human IL-3	Peprotech	Cat#:200-03
Recombinant human IL-7	Peprotech	200-07
Recombinant human SCF	Peprotech	Cat#:AF-300-07
Recombinant human TPO	Peprotech	Cat#:300-18
RPMI 1640	PAN Biotech	Cat#:P04-16500
SFEM II stem pan medium	Stemcell Technologies	Cat#:09605
sodium pyruvate	Anprotech	Cat#:P04-43100
Stem Regenin 1	Biogems	Cat#:1224999
TLR7 agonist RO7117419	Roche Diagnostics	N/A
Viastain AO staining solution	Nexcelom Biosciences	Cat#:CS2-0106
Zombie UV fixable viability kit	Biolegend	Cat#:423107
Critical commercial assays		
Chromium next GEM automated single cell 5’ Kit v2, module 1	10x Genomics	Cat#:1000292
Chromium next GEM automated single cell 5’ Kit v2, module 2	10x Genomics	Cat#:1000295
Chromium next GEM automated single cell 5’ Kit v2, module 3	10x Genomics	Cat#:1000294
Cytokine & chemokine 34-plex human ProcartaPlex panel	Thermo Fisher	Cat#:EPX340-12167-901
EasySep human NK cell enrichment kit	Stemcell Technologies	Cat#:19055
Foxp3 staining buffer set	BD Biosciences	Cat#:562725
IL-29 IFN lambda 1 human ProcartaPlex simplex kit	Thermo Fisher	Cat#:EPX01A-12049-901
Human CD8/NK Legendplex assay	Biolegend	Cat#:741187
Next GEM chip K & gaskets automated single cell	10x Genomics	Cat#:PN-1000297
Pan-IFN-α ELISA	PBL Assay Science	Cat#:41115-1
Pan-DC enrichment kit	Miltenyi Biotec	Cat#:130-100-777
Plasmacytoid dendritic cell isolation kit II, human	Miltenyi Biotec	Cat#:130-097-415
ProcartaPlex human basic kit	ThermoFisher	Cat#:EPX010-10420-901
Experimental models: Cell lines		
MS-5	DSMZ	RRID: CVCL_2128
Software and algorithms		
Bio-Plex manager v6.2.0.175	Bio-Rad	https://www.bio-rad.com/de-de/category/bio-plex-software
Besca package v2.4	Roche Diagnostics	https://bedapub.github.io/besca/
Bioconductor v3.15	Bioconductor	https://bioconductor.org/
Cell ranger single cell v6.0.2	10x Genomics	https://www.10xgenomics.com/support/software/cell-ranger/downloads
EdgeR v3.38.0, v3.40.2	Bioconductor package	https://bioconductor.org/packages/release/bioc/html/edgeR.html
Fgsea v1.24.0	Bioconductor package	https://bioconductor.org/packages/release/bioc/html/fgsea.html
FlowJo v10.8.1.	BD Biosciences	https://www.flowjo.com/solutions/flowjo/downloads
ggplot2 v3.3.6	R package	https://github.com/tidyverse/ggplot2
Graph Pad Prism v8	GraphPad Software	https://www.graphpad.com/
GSVA v1.46.0	Bioconductor package	https://bioconductor.org/packages/release/bioc/html/GSVA.html
pheatmap v1.0.12	R package	https://CRAN.R-project.org/package=pheatmap
UpSetR v1.4.0	R package	https://CRAN.R-project.org/package=UpSetR
R v4.2.0	Microsoft Corporation	https://www.R-project.org/

#### 2.1.2 Experimental model and cancer tissue details

##### 2.1.2.1 Murine MS-5 bone marrow stromal cell

Obtained from DSMZ (RRID: CVCL_2128) and cultured in MS-5 medium.

##### 2.1.2.2 Human CD34^+^ HSCs, peripheral blood mononuclear cells, and patient tumor samples

Cryopreserved human cord blood CD34^+^ hematopoietic stem and progenitor cells (HSCs) were purchased from Lonza. Peripheral blood mononuclear cells (PBMCs) were isolated from blood of anonymized healthy donors collected at the Medical Service of Roche Diagnostics GmbH, Penzberg. Single cell suspension from tumors or tumor tissues were obtained from Discovery Life Sciences, Fidelis and Indivumed either fresh shipped at 4°C in MACS tissue storage solution (Miltenyi Biotec) or frozen in pZerve freezing buffer (Sigma-Aldrich).

##### 2.1.2.3 Media

MS-5 cells were cultivated in MS-5 medium: MEM-alpha (Gibco) supplemented with 10% heat-inactivated FCS, 2% sodium pyruvate, 1% L-glutamine, 1% penicillin/streptomycin (all Anprotech). CD34^+^ HSCs were expanded in expansion medium: SFEM II stem pan medium (Stemcell Technologies) supplemented with 10% heat-inactivated FCS, 1% sodium-pyruvate, 1% penicillin/streptomycin (all Anprotech), 100 ng/mL FLT3-L, 100 ng/mL SCF, 50 ng/mL TPO, and 5 ng/mL IL-7 (all Peprotech). Expanded CD34^+^ HSCs were further differentiated in differentiation medium: MS-5 medium supplemented with 100 ng/mL FLT3-L, 100 ng/mL SCF, 5 ng/mL IL-7 (Peprotech), 0.5 mg/mL ascorbic acid (AA; Merck Millipore), 0.1 ng/mL GM-CSF (Biolegend), and 2.3 mM StemRegenin 1 (SR-1; Biogems). For assays involving primary or CB-pDCs, cells were cultivated in pDC medium: RPMI 1640 supplemented with 1% MEM non-essential amino acids, 1% MEM vitamin solution, 1% hepes buffer (all PAN Biotech), 10% heat-inactivated FCS, 1% sodium pyruvate, 1% L-glutamine, 1% penicillin/streptomycin (all Anprotech), 1% 2-Mercaptoethanol (Gibco).

### 2.2 Method details

#### 2.2.1 Generation of CB-pDCs from CD34^+^ HSCs

Cryopreserved human cord blood CD34^+^ HSCs were used for the generation of CB-pDCs. HSCs were thawed and 10^6^ cells were seeded at a density of 5,000 cells per well in 96-U-bottom plates (Corning) and expanded for 7 days in expansion medium at 37°C, 5% CO_2_, 5% O_2_. Expanded HSCs were harvested and counted before freezing [4x10^6^ cells/mL in freezing medium [90% heat-inactivated FCS (Anprotech) and 10% DMSO (Sigma-Aldrich)]. For differentiation of CB-pDCs from expanded HSCs, a feeder layer of MS-5 stromal cells (DSMZ) was prepared: MS-5 cells were thawed and passaged at least three times in MS-5 medium before treatment with 10 µg/mL Mitomycin C (Sigma-Aldrich) for 3 hrs. Treated MS-5 cells were harvested, washed and 25,000 cells per well were plated in 96-flat-bottom plates (Corning). The next day, 12,500 expanded HSCs were thawed and added onto a MS-5 feeder cell monolayer in differentiation medium. On day 7, fresh soluble factor cocktail was added. At day 12, differentiated cells were harvested, counted and used for subsequent analyses.

#### 2.2.2 Isolation of primary cells from blood

PBMCs were isolated from blood of healthy donors (freshly collected in heparin-containing NaCl from Medical Services of Roche Diagnostics GmbH, Penzberg) by centrifugation in Pancoll-filled Leucosep tubes (PAN Biotech). The PBMC layer was collected, washed in RPMI 1640 medium (PAN Biotech), centrifuged, re-suspended in RPMI 1640 medium and counted. PBMCs were used for enrichment of different immune cell populations as indicated.

PDCs were negatively enriched from PBMCs using the pDC isolation kit II (Miltenyi Biotec) according to the manufacturer’s instructions. pDC purity was assessed by CD123 and CD303 staining by flow cytometry and cells were used for subsequent assays.

NK cells were purified from PBMCs using Easy Sep Human NK cell enrichment kit (Stemcell Technologies) according to the manufacturer’s manual. Enriched NK cells were counted and checked for purity using CD3 and CD56 staining by flow cytometry before use in the co-culture assays.

Human pan-DCs (cDC1s, cDC2s, and pDCs) were enriched from PBMCs using an untouched human pan-DC enrichment kit (Miltenyi Biotec) according to the manufacturer’s instructions. The content of the different DC population was assessed from Living^+^CD45^+^Lin^-^ by CD123 and CD303 for pDCs, CD141 and CLEC9A for cDC1, and CD1c^+^ and CD11c^+^ for cDC2 staining by flow cytometry.

#### 2.2.3 IFN priming and TLR stimulation

For priming, 10,000 unsorted CB-pDCs (normalized on pDC % as assessed prior to the stimulation by flow cytometry) or 10,000 sorted CB-pDCs (10,000 pDCs/well) were plated in 96-U-bottom plates with pDC medium and 10 ng/mL IL-3 (Peprotech). IFN-β and IFN-γ (Peprotech) were titrated and added at 1 µg/mL, 100 ng/mL, 10 ng/mL, or 1 ng/mL for 72 hrs (IFN priming), with a final working concentration of 10 ng/mL for all experiments. Following IFN priming, cells were washed 3 times with pDC medium to remove remaining IFN and were used for stimulation with TLR agonists.

For stimulation of primed CB-pDCs and primary pDCs, 10,000 pDCs/well were plated in 96-U-bottom plates in pDC medium. For stimulation, CpG-A ODN 2216 (TLR9 agonist, Invivogen) was used at 5 µg/mL, R848 (TLR7/8 agonist, Invivogen) at 285 nM (100 ng/mL) and RO7117419 (TLR7, Roche proprietary molecule, U.S. patent US2020268762A1, 2020) at 1 µM final concentration for 2 hrs, 4 hrs, or 24 hrs as indicated.

#### 2.2.4 pDC-NK cell co-culture

Primed CB-pDCs and primary pDCs were plated in 96-U-bottom plates in pDC medium and stimulated with TLR agonists as described above. After 2 hrs, primary NK cells were added at a 5:1 ratio (1x10^4^ CB-pDCs : 5x10^4^ NK cells). After 24 hrs of co-culture, supernatant was collected for cytokine and chemokine analysis and cells were stained for flow cytometry.

#### 2.2.5 Flow cytometry and sorting

For live/dead cell discrimination, cells were resuspended in 100 µL/well of Zombie UV Fixable viability kit (Biolegend; diluted 1:400) containing 5 µL/well human Trustain FcX (Biolegend) for 20 min at 4°C in the dark. For surface staining, cells were resuspended in 50 µL surface staining master mix containing antibodies (**Table 1**) diluted in FACS buffer and incubated for 20 min at 4°C in the dark. Cells were washed, resuspended in 150 µL FACS buffer and acquired on a BD LSRFortessa or Cytek Aurora, respectively. For cytokines and transcription factors, intracellular staining was performed using the FOXP3 staining buffer set (BD Biosciences) according to manufacturer’s instructions on cells previously stained for extracellular membrane antigens.

For sorting, CB-pDCs were incubated with a sorting master mix for 20 min at 4°C on day 12 of the *in vitro* differentiation. CB-pDCs were sorted on a BD FACSAria Fusion Cell Sorter with a 100 micron nozzle. In brief, morphology and single cell gating was used to exclude debris, big cells and doublets, CB-pDCs were defined as CD45^+^Lineage^-^CD123^+^CD45RA^+^AXL^-^CD327^-^ cells. Sorted cells were counted using a ViCell analyzer before plating. The detailed sorting strategy is shown in **Supplementary Figures S3A, B**.

#### 2.2.6 Human tumor specimen

Single cell suspension from tumors was obtained from Discovery Life Sciences. Primary human tumor tissue was obtained from Fidelis or Indivumed either fresh shipped at 4°C in MACS tissue storage solution (Miltenyi Biotec) or frozen in pZerve freezing buffer (Sigma-Aldrich). Tumor tissues were minced in small pieces with scissors or scalpels and incubated with a digestion buffer containing 1 mL MACS tissue storage solution (Miltenyi Biotec), 1 mL Accutase (PAN Biotech), 1% BSA (Sigma-Aldrich), 275 U/mL Collagenase IV (Worthington), 10 U/mL DNase I type 4 (Sigma-Aldrich), 471 U/mL Hyaluronidase (Sigma-Aldrich) for 30 min at 37°C at 200 rpm. To stop the enzymatic reaction, RPMI 1640 containing 2% FCS was added and digested tumor pieces were mashed onto a 100 µM filter. The single cell suspension was spun down at 300x g for 15 min, resuspended in RPMI 1640 and counted using a ViCell analyzer. Next, cells were spun down again and used in subsequent experiments or resuspended in the pZerve freezing buffer for long term storage.

#### 2.2.7 Co-culture of CB-pDCs and single cell suspension from digested tumors

Sorted and primed CB-pDCs were plated in pDC medium and stimulated with TLR agonists as described above for 2 hrs. In parallel, tumor digests were prepared from frozen tumor tissue as described above or frozen tumor digests from digested tumor tissues were thawed, counted and spun down for 10 min at 400x g. Cells from tumor cell suspension were plated in a 96-U-bottom plate in pDC medium with 20 ng/mL IL-2 (Roche) and 10 ng/mL IL-15 (Peprotech) and stimulated CB-pDCs were added at 1:10 ratio (3x10^4^ CB-pDCs : 3x10^5^ cells of tumor cell suspension) or incubated with 1 µM TLR7 agonist (RO7117419). After 24 hrs, supernatants were collected for cytokine analysis.

#### 2.2.8 Cytokine and chemokine analysis

For detection of most IFNa subtypes, a pan-IFNa ELISA (PBL Biosciences) was performed according to the manufacturer’s manual. Absorbance was measured at 450 nm on a Tecan infinite microplate reader.

To detect various cytokines, chemokines and soluble factors, multiplex cytokine assays were performed. Supernatants were mixed at 500 rpm with analyte-specific pre-coated beads for 2 hrs at RT (Thermo Fisher, Bio-Rad). Next, samples were incubated with analyte-specific biotinylated antibodies for 30 min. Finally, PhycoErythrin (PE) was added to reveal the bead-analyte-detection antibody scaffold. Samples were measured on a Bio-Plex 200 analyzer and results were analyzed using the Bio-Plex manager software. For analysis of soluble cytotoxic mediators the Human CD8/NK Legendplex assay (Biolegend) was performed according to the manufacturer’s instructions and samples were analyzed on a LSRFortessa (BD Biosciences).

#### 2.2.9 Single cell RNA sequencing

##### 2.2.9.1 Library preparation and sequencing

For single cell RNA (scRNA) sequencing, differentiated CB-DCs (3 independent donors) either left unprimed or primed with IFN were used to enable characterization of the heterogeneity of the *in vitro* differentiation protocol. For comparison, primary pan-DCs (3 independent donors) were isolated from PBMCs as described above. CB-DCs and primary pan-DCs were normalized to 10,000 pDCs per well and stimulated with TLR9 or TLR7 agonists for 4 hrs or left untreated. In total 27 samples were analyzed (**Table 2**).

**Table 2:** Overview of samples included in the scRNA sequencing experiment.

Sample name	Number of replicates (different donors)	Description
Pan-DCs untreated	3	Pan-DCs enriched from PBMCs, left untreated.
Pan-DCs TLR9 treated	3	Pan-DCs enriched from PBMCs, treated with a TLR9 agonist for 4 hrs.
Pan-DCs TLR7 treated	3	Pan-DCs enriched from PBMCs, treated with a TLR7 agonist for 4 hrs.
CB-DCs unprimed untreated	3	Differentiated CB-DCs were left unprimed for 72 hrs and next, left untreated.
CB-DCs unprimed TLR9 treated	3	Differentiated CB-DCs were left unprimed for 72 hrs and next, treated with a TLR9 agonist for 4 hrs.
CB-DCs unprimed TLR7 treated	3	Differentiated CB-DCs were left unprimed for 72 hrs and next, treated with a TLR7 agonist for 4 hrs.
CB-DCs unprimed untreated	3	Differentiated CB-DCs were primed for 72 hrs and next, left untreated.
CB-DCs unprimed TLR9 treated	3	Differentiated CB-DCs were primed for 72 hrs and next, treated with a TLR9 agonist for 4 hrs.
CB-DCs unprimed TLR7 treated	3	Differentiated CB-DCs were primed for 72 hrs and next, treated with a TLR7 agonist for 4 hrs.

ScRNA sequencing was performed using Chromium Connect (10x Genomics). Next GEM Automated Single Cell 5’ Reagent Kits v2 (PN-1000290, 10x Genomics) were used following the manufacturer’s protocol. Roughly 8,000–10,000 cells per sample were diluted at a density of 100–800 cells/μL in PBS plus 1% BSA determined by Cellometer Auto 2000 Cell Viability Counter (Nexelom Bioscience), and were loaded onto the chip. The quality and concentration of both cDNA and libraries were assessed using an Agilent BioAnalyzer with High Sensitivity DNA kit (#5067–4626, Agilent) and Qubit Fluorometer with Qubit dsDNA HS assay kit (#Q33230, Thermo Fisher) according to the manufacturer’s recommendation. For sequencing, samples were mixed in equimolar fashion and sequenced on an Illumina Nova Seq 6000 with a targeted read depth of 20,000 reads/cell and sequencing parameters were set for Read 1 (26 cycles), i7 Index (10 cycles), i5 Index (10 cycles) and Read 2 (90 cycles). The Cell Ranger mkfastq function was used to convert the output files into FASTQ files.

##### 2.2.9.2 Computational analysis

For pre-processing and quality control, raw sequencing reads were de-multiplexed and mapped to the *GRCh38* genome using the Cell Ranger Single Cell software (10x Genomics). Raw gene expression matrices generated per sample were merged and analyzed with the besca package (35). First, low quality cells and potential multiplets were excluded (minimum 600 genes, 1,000 counts, maximum 6,500 genes and 60,000 counts), resulting in 4,000 to 8,000 cells per sample and a total of 183,398 cells passing quality control for downstream analysis. Filtered cells were normalized by log-transformed UMI counts per 10,000 reads [log(CP10K+1)]. After scaling the gene expression, the most variable genes per sample were calculated (minimum mean expression of 0.0125, maximum mean expression of 3 and minimum dispersion of 0.5) and those shared by at least 50% of the samples, in total 2,208 genes, were used for principal component (PC) analysis. Finally, the first 50 PCs were used as input for calculating the 10 nearest neighbors and the neighborhood graph was then embedded into the two-dimensional space using the uniform manifold approximation and projection (UMAP) algorithm (36). Cell clustering was performed using the Leiden algorithm (37).

##### 2.2.9.3 Cell type annotation

Annotation was performed using the Sig-annot semi-automated besca module. The gene sets used for different cell types can be found under https://github.com/bedapub/besca/blob/main/besca/datasets/genesets/CellNames_scseqCMs6_sigs.gmt. First, each cluster was assigned to a cell type at different levels of granularity. Subsequently, annotations were manually inspected to resolve cluster mixtures, especially for different DC types. Cell type annotations were further curated by selecting a cluster and applying heuristic cutoffs on a combination of signature scores to reannotate individual cells. The per-cell signature scores were calculated with the scanpy function scanpy.tl.score_genes, using default parameters and besca signatures. Cells annotated as doublets were excluded from downstream analyses. In order to generate visualizations, such as the expression level of selected genes across conditions, custom scripts with mainly besca and scanpy functions were used.

##### 2.2.9.4 Differential gene expression (DGE) analysis

PDCs were selected and summed into pseudobulks by sample. The analysis followed the Orchestrating Single-Cell Analysis with Bioconductor guidelines using edgeR (38, 39). To extract the treatment effect, CB and pan-DC samples were separated and analyzed individually. For CB samples, a treatment*primer + donor design was chosen, while for pan-DCs, treatment + donor was selected since no priming was performed here. To extract the tissue effect (CB vs. pan-DCs) all samples were combined and a tissue_primer*treatment + W_1 + W_2 was chosen, where tissue_primer has three levels: pan-DCs, CB_unprimed, CB_primed. W_1 and W_2 are covariates to remove unwanted variation by adjusting for nuisance effects through factor analysis on samples combined, grouped by tissue, treatment and priming (40). This models the donor effect that cannot be directly included in this case, because pan-DCs and CB samples are from different donors. The visualizations were generated using ggplot2, pheatmap and UpSetR in R (41).

##### 2.2.9.5 Gene set enrichment analysis (GSEA)

GSEA enrichment of previously reported DC subset-specific signatures in unprimed and untreated pDCs was assessed using single sample gene set enrichment analysis, as implemented in the gene set variation analysis (GSVA) Bioconductor package (42). Briefly, gene signatures were obtained from Villani et al. and pseudobulk raw counts were normalized for differences in library size using edgeR (38, 39). Enrichment of 6 different DC signatures (as reported by Villanni et al.) was calculated per pseudobulk using default settings in GSVA, and visualized using pheatmap (43).

##### 2.2.9.6 BubbleGUM visualization

To explore the list of differentially expressed genes, GSEA was further used as implemented in fgsea and various gene set collections from the molecular signature database as previously described (44–46). To condense and visualize several GSEA runs, GSEA unlimited map (BubbleGUM) plots were selected as previously described (47). Starting with the set of genes shared among the selected DEG tables, the product of logFC and -log10(PValue) was defined as gene ranking metric, and fgsea ran with 1E5 permutations and other default settings. The resulting normalized enrichment score (NES) and adjusted P values were then extracted from fgsea output tables and visualized using ggplot2.

#### 2.2.10 Statistical analysis

Statistical analysis was performed using Prism 8 software (Graph Pad). Data were tested for normal distribution using the Shapiro-Wilk test. For data following normal distribution, paired Student’s t-test, one way ANOVA or two way ANOVA with Tukey *post-hoc* test were performed. For non-normal distributed data, the Wilcoxon test or the Kruskal-Wallis test with a Dunn’s *post-hoc* test was performed. All statistical tests performed as well as biological replicates are indicated in the legends of the corresponding figures. Statistical significances between groups were represented as follow: ns: non significant; *, p<0.05; **, p<0.01; ***, p<0.001; ****, p<0.0001.

#### 2.2.11 Illustrations

Illustrations were created using Biorender.com.

#### 2.2.12 Data availability statement

This paper does not report original code. The raw and processed scRNAseq datasets can be found at https://doi.org/10.6084/m9.figshare.25561950.v1. Any additional information required to reanalyze the data reported in this paper is available upon request.

## 3 Results

### 3.1 Tumor-associated pDCs fail to respond to TLR stimulation

**Figure 1:**
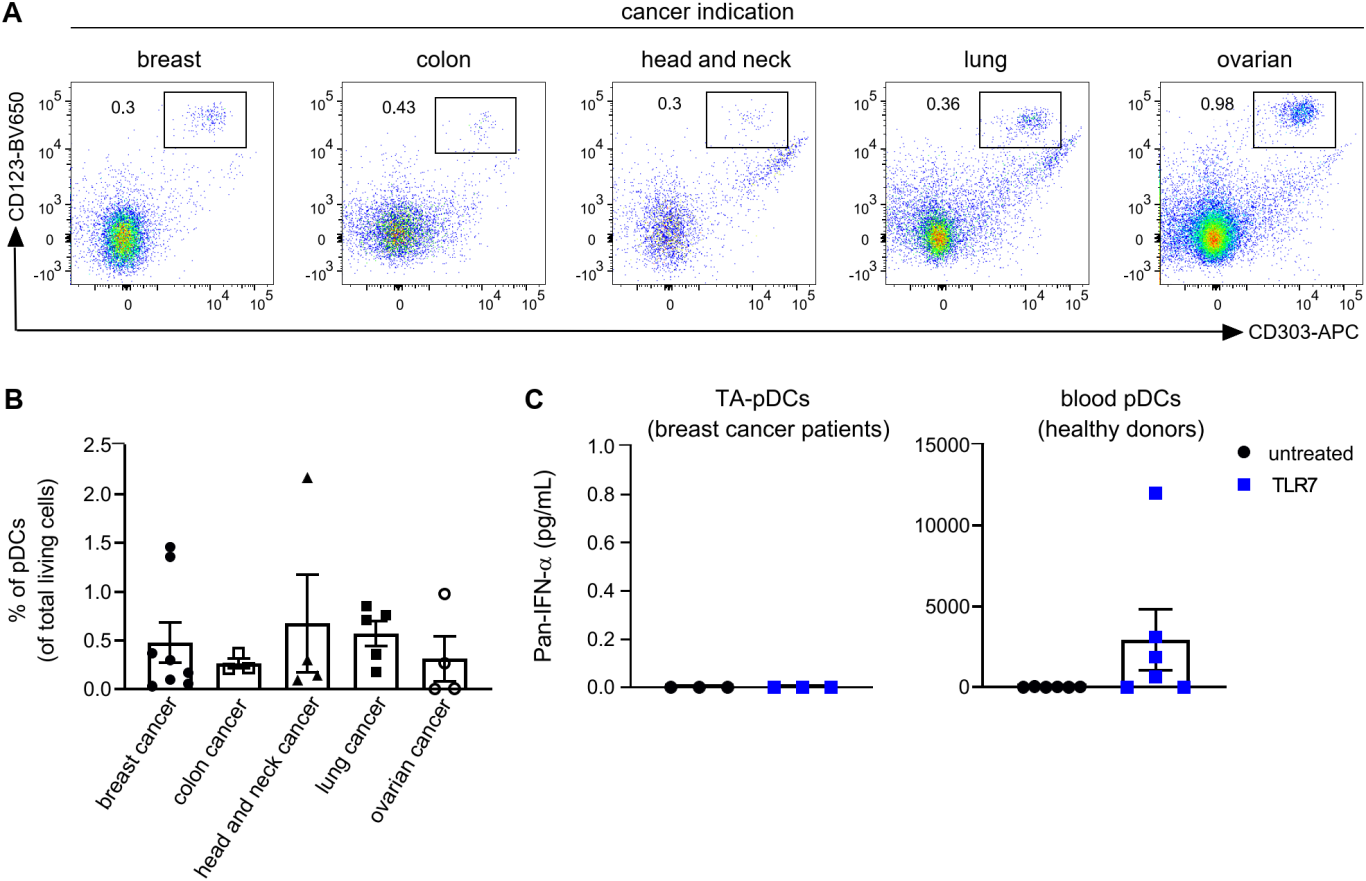
Tumor-associated pDCs are tolerogenic towards stimulation with TLR7 agonist. **(A)** Representative dot plots showing TA-pDCs infiltrating various solid tumor types as detected by CD123^+^CD303^+^ staining in single cell suspensions from tumor digests using flow cytometry. **(B)** Quantification of TA-pDCs (defined as Lin^-^CD123^+^CD303^+^ cells) as fraction of living cells in various tumor indications (breast cancer: n=8; colon cancer: n=3; lung cancer: n=4; head and neck cancer: n=5; ovarian cancer: n=4). **(C)** Quantification of IFN-ɑ release from TLR7-stimulated (left) single cell suspension from breast cancer tissue (n=3) and blood pDCs (right) from healthy donors (n=6). Results are shown as mean ± SEM. See also **Supplementary Table S1**.

With the aim to investigate the potential of pDCs for cancer immunotherapy, we first determined the ability of TA-pDCs to respond to TLR stimulation. Tumor digests from breast, colon, head and neck, lung, and ovarian cancer patients were screened for their TA-pDC content using flow cytometry based on expression of CD123 and CD303 (**Figure 1A** and **Supplementary Table S1**). Scarce amounts of TA-pDCs were identified within the samples representing up to 2% of living cells. Across indications, the highest TA-pDC content was detected in breast and lung cancer samples (**Figure 1B**). Subsequently, tumor digests of breast cancer samples with solid pDC infiltration and whole blood samples of healthy donors were stimulated with a TLR7 agonist (RO7117419) for 24 hrs (**Figure 1C**). Upon stimulation, TA-pDCs failed to secrete IFN-α contrary to blood pDCs from healthy donors. In this specific context, stimulation of TA-pDCs with a TLR7 agonist appeared to be challenging, therefore a different approach leveraging the potential of pDCs might be more suitable for cancer immunotherapy.

### 3.2 Generation of high yields of *in vitro* differentiated CB-pDCs

Instead of focusing on *in situ* activation of immunosuppressive TA-pDCs, we hypothesized that pre-activated pDCs could be used as cell therapy to trigger inflammation in the TME. As pDCs are rare in peripheral blood, we selected CB-pDCs as an alternative source of cells and aimed at developing an *in vitro* differentiation protocol tailored to support the generation of large numbers of pDCs that could directly be used as cancer immunotherapy.

**Figure 2:**
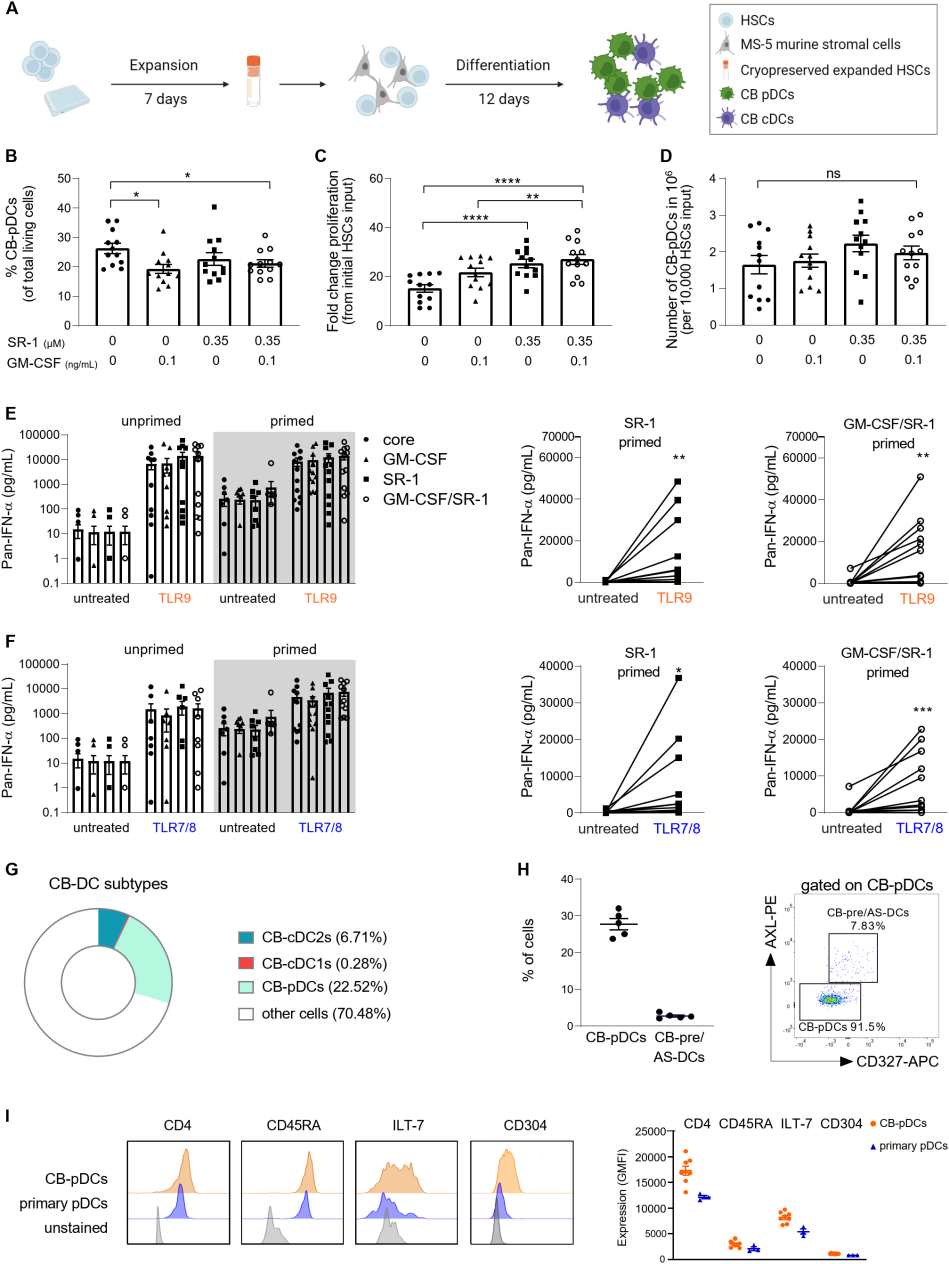
Generation of *in vitro* differentiated CB-pDCs from CD34^+^ HSCs. **(A)** Schematic overview depicting the generation of CB-pDCs from CB-derived CD34^+^ HSCs. 5,000 HSCs were expanded for 7 days in expansion media and cryopreserved. Expanded, thawed HSCs were differentiated for 12 days on a monolayer of Mitomycin C-treated MS-5 stromal cells before being harvested on day 19 or used for subsequent experiments. **(B)** Graph displaying the frequencies of CB-pDCs as percentage of living cells. **(C)** Graph displaying the total cell proliferation in the mixed culture during differentiation by fold increase and **(D)** absolute CB-pDC numbers corrected on the frequency of differentiated CB-pDCs on day 19 comparing supplementation of SR-1 and GM-CSF separately or in combination. **(E, F)** Quantification of IFN-ɑ release from **(E)** TLR9-activated and **(F)** TLR7-activated cells in the mixed culture comparing unprimed and primed cells of the different conditions with LLOD at 1.95 pg/mL. **(G)** Pie chart showing the mean frequencies of CB-cDC1s, CB-cDC2s, CB-pDCs, and other not further characterized cells from 6 independent CB donors as defined by flow cytometry phenotyping. **(H)** Quantification of CB-pDCs and CB-pre/AS-DC frequencies (from CD45^+^Lin^-^CD123^+^CD45RA^+^) from 5 independent CB donors and dot plot displaying CB-pDC and CB-pre/AS-DC gates from one representative donor. **(I)** Overlaid histograms comparing expression of CD4, CD45RA, ILT-7, and CD304 of CB-pDCs and primary pDCs of one representative donor. Graph depicts GMFI of CD4, CD45RA, ILT-7, and CD304 as determined by flow cytometry shown as mean ± SEM of 2 independent experiments, 8 independent CB donors, and 3 independent pDC donors. **(B–F)** Results are shown as mean ± SEM of 4 independent experiments and 12 independent CB donors. *p<0.05; **p<0.01, ***p<0.001, ****p<0.0001, ns: not significant **(B–D)** one-way ANOVA with Tukey’s *post-hoc* test or **(E, F)** Wilcoxon test. See also **Supplementary Figure S1**.

First, we tested various conditions according to previously published protocols. To increase the overall yield, CD34^+^ HSCs were expanded for 7 days with FLT3-L, SCF, TPO, and IL-7 as previously described (32). Expanded CD34^+^ HSCs were differentiated on MS-5 stromal cells for 12 days with FLT3-L, SCF, IL-7, and ascorbic acid as a baseline soluble factor cocktail (**Figure 2A**). Additionally, GM-CSF and StemRegenin 1 (SR-1) were shown to promote CB-pDC differentiation (27, 30, 33).

Thus, we tested if GM-CSF and SR-1 alone or in combination would increase pDC yield during the differentiation phase (**Figures 2B–F** and **Supplementary Figure S1A**). The percentage of CB-pDCs was evaluated based on expression of CD123 and CD303 (**Figure 2B** and **Supplementary Figure S1B**). Addition of GM-CSF and SR-1 alone or in combination negatively regulated CB-pDC differentiation as reflected by a decrease in CB-pDC frequencies from 26% to 20.8%. On the contrary, both factors promoted proliferation in the mixed culture resulting in increased absolute numbers of CB-pDCs for cells exposed to SR-1 alone or in combination with GM-CSF reaching around 2x10^6^ CB-pDCs per 10,000 HSCs input (**Figures 2B–D** and **Supplementary Table S2**).

Next, the functionality of CB-pDCs was tested by measuring IFN-ɑ secretion as a proxy for CB-pDC activation in the mixed cultures following TLR9 (CpG-A) or TLR7/8 (Resiquimod) agonist stimulation (**Figures 2E, F**). However, TLR stimulation alone failed to elicit a robust IFN-ɑ response in TLR7/8-stimulated mixed cells (**Figure 2F**). This limitation of *in vitro* differentiated pDCs was previously described by Laustsen et al. reporting that the treatment with IFN-β and IFN-ɣ prior to TLR stimulation enables IFN-ɑ release (33). Thus, following *in vitro* differentiation, cells were exposed to IFN-β and IFN-ɣ for 72 hrs, a step called IFN priming. Upon IFN priming and TLR stimulation, *in vitro* differentiated cells exposed to SR-1 and the combination of GM-CSF and SR-1 exhibited a slight increase in IFN-ɑ release compared to other conditions, thus suggesting that both soluble factors together can promote CB-pDC functionality.

This protocol using FLT3-L, SCF, IL-7, ascorbic acid, GM-CSF, SR-1, and MS-5 stromal cells to support differentiation yielded 2x10^8^ CB-pDCs on average from 10^6^ HSCs and elicited the strongest functionality. Therefore, this factor combination was selected for further experiments.

Most soluble factors supporting CB-pDC differentiation are also described to promote differentiation of conventional DCs and their precursor population pre/AS-DCs (31, 48). To characterize the cell composition more in depth, cells were phenotyped and all three main DC subtypes were identified, however a skewing towards pDC differentiation was observed (**Figure 2G** and **Supplementary Figures S1C**, **E**). Pre/AS-DCs are described as a cDC precursor population expressing CD123 and CD303 (43, 49). Similar to observations with primary pDCs, a small pre/AS-DCs population expressing AXL and CD327 was detected by flow cytometry within the CB-pDC gate (**Figure 2H**). Overall, the DC compartment encompassing all main DC subtypes accounted for about 30% of all cells highlighting the versatility of this *in vitro* differentiation protocol. In addition, the CB-pDC population partly expressed CD11c but to a lower extent compared to cDC2s (**Supplementary Figure S1D**).

Finally, the phenotype of CB-pDCs was further examined. Besides the canonical markers CD303 and CD123, human pDCs also express the surface markers CD4, CD45RA, ILT-7, and CD304 (1, 3). Their expression levels were compared between CB-pDCs and primary pDCs by flow cytometry. CB-pDCs recapitulate the expression of all markers in a similar fashion as their blood counterparts (**Figure 2I** and **Supplementary Figure S1F**).

Taken together, we describe an *in vitro* differentiation protocol tailored to support the large scale differentiation of functional human CB-pDCs phenotypically resembling primary pDCs.

### 3.3 CB-pDCs transcriptionally resemble primary pDCs

With the aim to comprehensively compare CB- and primary pDCs, we performed scRNA sequencing. The transcriptome of all *in vitro* differentiated cells was analyzed to avoid selection bias from phenotypic markers. *In vitro* differentiated cells were compared to primary pan-DCs enriched from PBMC in order to assemble the frequencies of the different DC subtypes (**Supplementary Figure S2A** and **Figure 2G**).

**Figure 3:**
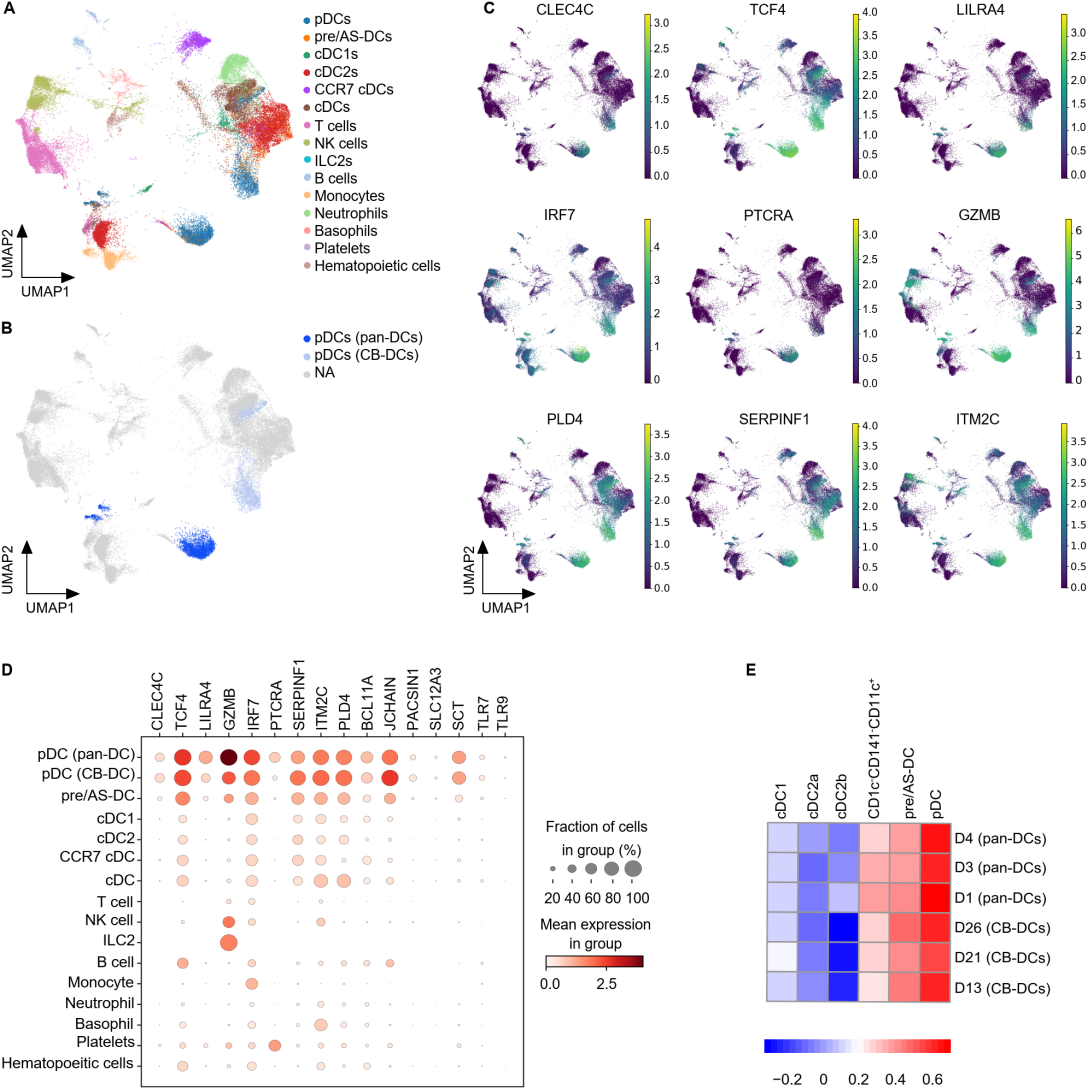
CB-pDCs resemble primary pDCs at the transcriptional level. **(A)** UMAP plot showing the immune cell subsets in CB-DCs and primary pan-DCs. **(B)** UMAP plot showing pDCs resolved from enriched pan-DC and CB-DC samples. **(C)** UMAP plot showing the gene expression levels of different pDC markers. NA stands for not applicable and refers to cells not annotated as pDCs due to the lack of pDC marker expression. **(D)** Mean expression levels of selected pDC signature genes in the different immune cell subsets. Dot size indicates fraction of positive cells, color indicates mean expression levels. **(E)** GSEA analysis comparing CB-DCs of the different donors with the DC signatures published by Villani et al. Results of 3 independent untreated, unprimed CB donors and 3 independent pan-DC donors are depicted. See also **Supplementary Figure S2**.

The *in vitro* differentiated cells appeared clearly segregated from pan-DC samples and both exhibited a very heterogeneous composition (**Supplementary Figures S2B**, **C**). As expected, the DC compartment was strongly represented (**Figure 3A** and **Supplementary Figure S2C**). Lymphoid cell types such as T and NK cells were mainly present in pan-DC samples whereas CB samples also contained neutrophils and basophils confirming the strong myeloid bias of the *in vitro* differentiation protocol (**Supplementary Figure S2C**). PDC clusters were observed in CB- and pan-DC samples and evaluated for their expression of previously described pDC genes (26, 43) (**Figure 3B** and **Supplementary Figure S2D**). CB-pDCs recapitulated a canonical pDC gene expression pattern including the pDC master transcription factor *TCF4*, the surface markers *LILRA4*, *CLEC4C* (*CD303*), *PLD4*, and genes involved in pDC function such as *TLR9* and *TLR7*. The expression patterns of these genes were similar between CB-pDCs and primary pDCs with the exception of *IRF7, PTCRA* and *GZMB* that were either more strongly expressed or expressed in more cells for primary pDCs (**Figures 3C, D**, and **Supplementary Figure S2E**). Conversely, *IL3RA* was less expressed in CB-pDCs which could be attributed to a longer culture in presence of IL-3 compared to primary pDCs (**Supplementary Figure S2E**). Moreover, when compared to other immune cell types in this dataset, only CB-pDCs, primary pDCs, and pre/AS-DCs showed concomitant expression of all pDC-associated genes analyzed, yet the latter to a lower extent (**Figure 3D**).

To further characterize CB-pDCs, their transcriptional signature was compared to the 6 human circulating DC signatures published by Villani and colleagues (43). GSEA analysis confirmed that CB-pDCs were strongly enriched for the pDC signature, similar to primary pDCs in our dataset (**Figure 3E**). In addition, both populations showed, to a lesser extent, enrichment for the pre/AS-DC signature and poorer enrichment for cDC1, cDC2, and CD1c^-^CD141^-^CD11c^+^ signatures. Altogether, these data illustrate that *in vitro* differentiated CB-pDCs robustly resemble *bona fide* pDCs at the transcriptional level.

### 3.4 CB-pDCs and primary pDCs share key functional features

Next, we extensively characterized the functionality of sorted CB-pDCs in a head-to-head comparison with primary pDCs. We performed a titration of IFN-β and IFN-ɣ for the IFN priming step to determine the minimal IFN concentration enabling CB-pDC functionality. CB-pDCs were sorted and primed with decreasing concentrations of IFN-β and IFN-ɣ before stimulation with TLR9 (CpG-A) or TLR7 (RO7117419) agonists for 24 hrs (**Supplementary Figures S3A–C**). IFN-ɑ secretion was sustained upon IFN priming with 1 µg/mL and 10 ng/mL after TLR9 stimulation. Nevertheless, it appeared to be dose-dependent following TLR7 stimulation. Interestingly, the maturation markers CD40 and CD80 were up-regulated upon TLR9 or TLR7 stimulation independently of priming. Thus, 10 ng/mL of IFN-β and IFN-ɣ was selected for priming for further experiments (**Supplementary Figure S3C**).

**Figure 4:**
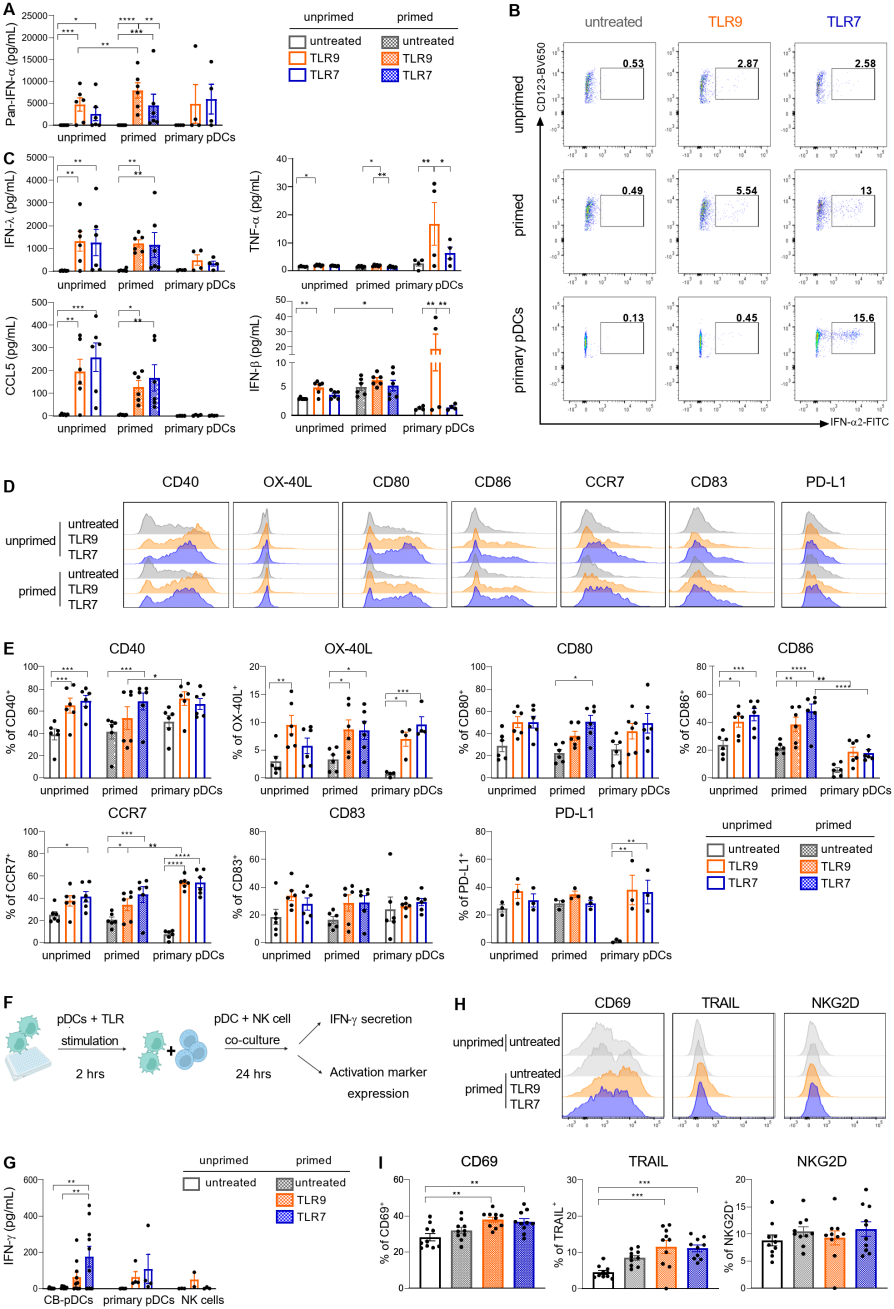
CB-pDCs show key functional features resembling primary pDCs. Unprimed and primed sorted CB-pDCs as well as primary pDCs were activated by TLR9 or TLR7 agonists for 24 hrs. **(A)** Quantification of pan-IFN-ɑ release in supernatant. **(B)** Representative flow cytometry dot plots showing expression of intracellular IFN-ɑ2 after 6 hrs (from CD45^+^CD123^+^CD303^+^) of 2 independent experiments. **(C)** Quantification of IFN-λ1, TNF-ɑ, CCL5, and IFN-β in supernatant. **(D)** Overlaid histograms comparing expression of activation markers on the surface of the different pDC types. **(E)** Quantification of marker expression on the surface of pDCs (from CD45^+^CD123^+^CD303^+^). **(F)** Scheme illustrating the set-up of pDC-NK cell co-culture assay. **(G)** Quantification of IFN-ɣ in supernatant upon co-culture of NK cells with CB-pDCs or pDCs. **(H)** Overlaid histograms showing activation marker expression on NK cells upon co-culture with CB-pDCs. **(I)** Quantification of activation marker expression on NK cells (from CD45^+^CD56^+^CD123^-^) upon co-culture with pDCs. Results are shown as mean ± SEM of 2 independent experiments, **(A, C, E)** 6 independent CB donors and 4 independent primary pDC donors and **(G, I)** 5 independent CB donors, 4 independent pDC donors and 4 independent NK cell donors are depicted. *p<0.05; **p<0.01, ***p<0.001, ****p<0.0001, **(A, C, E)** two-way ANOVA or **(G, I)** one-way ANOVA with Tukey’s *post-hoc* test. See also **Supplementary Figure S3**.

PDCs were extensively described to support inflammation and immune responses via the secretion of inflammatory cytokines including type I IFNs and chemokines as well as providing co-stimulation to various immune cell types (4, 50, 51). Upon TLR9 or TLR7 stimulation, primed CB-pDCs secreted comparable levels of IFN-ɑ as primary pDCs (**Figure 4A**). Moreover, IFN-ɑ2 could be detected intracellularly after 6 hrs in stimulated sorted CB-pDCs, with primed CB-pDCs and primary pDCs displaying an enhanced frequency of IFN-ɑ producing cells, especially upon TLR7 stimulation (**Figure 4B**). CB-pDCs also secreted higher amounts of several subtypes of type III IFN, such as IFN-λ1 and the chemokines CCL4 and CCL5 compared to primary pDCs (**Figure 4C** and **Supplementary Figure S3D**). Conversely, TLR9-treated primary pDCs exhibited a stronger IFN-β secretion. Stimulated CB-pDCs failed to produce TNF-ɑ and IL-6 contrary to their blood counterparts as well as IL-12p70 similarly to primary pDCs (**Figure 4C** and **Supplementary Figure S3D**). In addition, TLR9 and TLR7 stimulation elicited a strong upregulation of various co-stimulatory molecules including CD40, OX-40L, CD80, CD86, CD83, and the chemokine receptor CCR7 comparable to primary pDCs (**Figures 4D, E**). Although primary pDCs upregulated PD-L1 upon stimulation, CB-pDCs showed PD-L1 expression at baseline that could not be further modulated. Of note, induction of most of the cytokines and co-stimulatory molecules seemingly relied on stimulation rather than priming with the exception of IFN-α. Primary pDCs were reported to support NK cell activation as part of the innate immune system primarily via IFN-ɑ secretion (52, 53). With CB-pDCs demonstrating a highly functional phenotype in terms of cytokine release and maturation marker induction, we aimed to further study their functionality in a heterologous co-culture with NK cells. NK cell activation was evaluated after 24 hrs of co-culture with primed and TLR-stimulated CB-pDCs or TLR-stimulated primary pDCs (**Figure 4F**). NK cells cultivated in presence of TLR7-activated CB-pDCs displayed the strongest IFN-ɣ release compared to TLR9-stimulated CB-pDCs and TLR9- and TLR7-stimulated primary pDCs (**Figure 4G**). Furthermore, in presence of TLR9- and TLR7-treated CB-pDCs, NK cells up-regulated the activation marker CD69 and cytotoxic ligand TRAIL (**Figures 4H, I**). However, no modulation of NKG2D was observed. Taken together, these results show the ability of CB-pDCs to strongly respond to TLR stimulation, enabling the activation of innate effector cells and further substantiating their homology to human pDCs.

### 3.5 TLR stimulation activates similar genes and pathways in CB and primary pDCs

**Figure 5:**
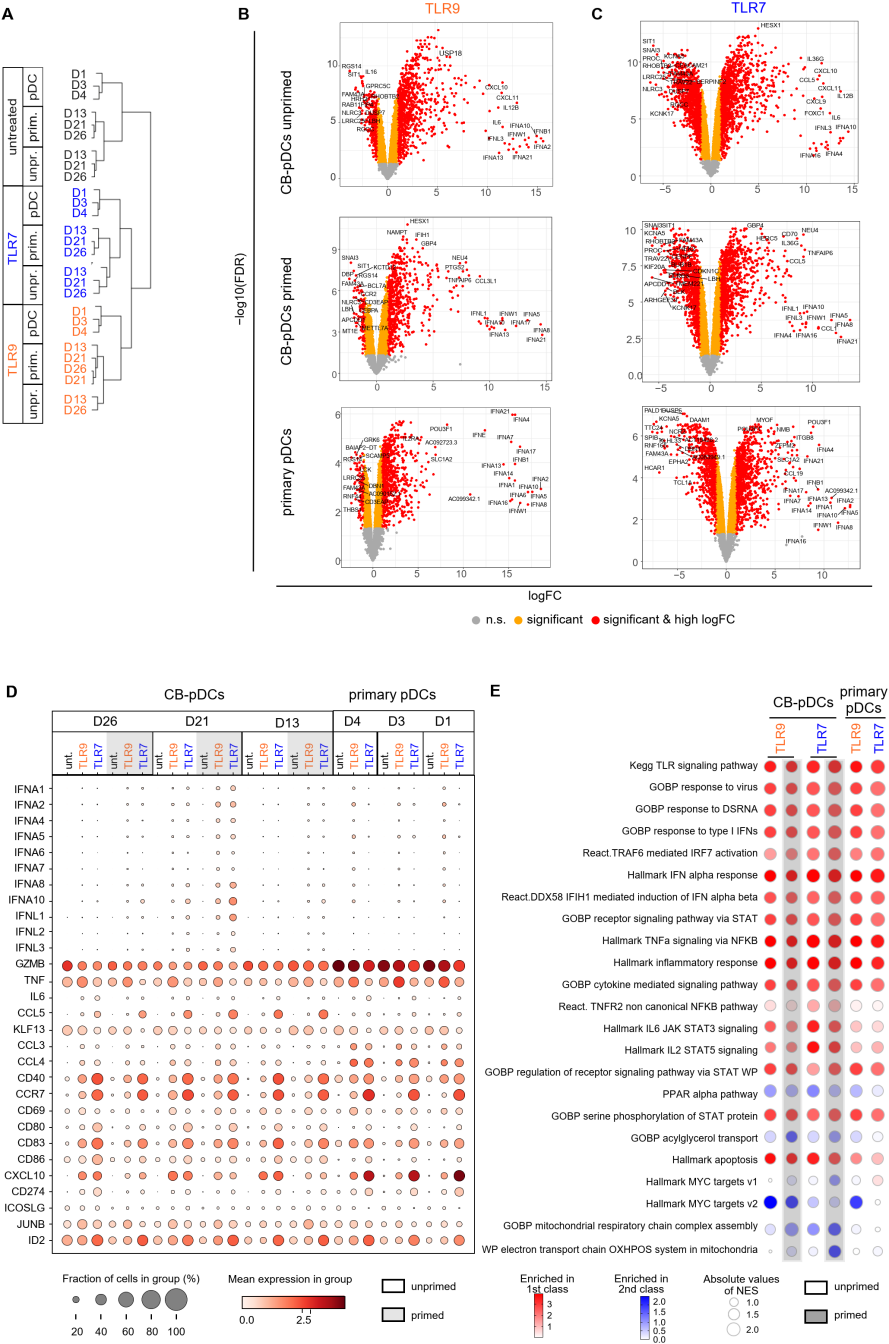
TLR stimulation activates similar genes and pathways in CB and primary pDCs. **(A)** Cluster dendrogram depicting a hierarchical clustering analysis of the different donors comparing primary pDCs to CB-pDCs with or without priming upon TLR stimulation or left untreated. **(B, C)** Volcano plots showing differentially expressed genes (right: up-regulated, left: down-regulated) between untreated and upon **(B)** TLR9 or **(C)** TLR7 activation of various pDC types as indicated by labeling. Colors display significance (orange) and significance and high log fold change (red), n.s.: not significant (gray). **(D)** Dot plot depicting the fraction of positive cells (dot size) and their mean expression levels (dot color) of different donors and various treatments as indicated. Genes were selected based on function or activation in pDCs. **(E)** BubbleMap showing the most relevant pathways enriched in TLR9- and TLR7-activated CB-pDCs and primary pDCs. Color code as explained in the figure, increasing intensity represents stronger enrichment in TLR-treated groups (red) or in untreated controls (blue) with bubble sizes corresponding to the absolute values for the normalized enrichment score. Results of 3 independent CB donors and 3 independent pan-DC donors are shown, with unprimed and primed CB cells and primary pan-DCs with or without activation with TLR9 or TLR7 agonists for 4 hrs. See also **Supplementary Figure S4** and **Supplementary Table S3**.

We next aimed to confirm these findings by comparing early transcriptional responses of stimulated CB- and primary pDCs by scRNA sequencing. Unprimed and primed *in vitro* differentiated cells as well as pan-DCs were stimulated with TLR9 or TLR7 agonists for 4 hrs before preparing for scRNA sequencing. We first performed a hierarchical clustering analysis to group samples and conditions based on their transcriptional resemblance (**Figure 5A** and **Supplementary Figure S4A**). This analysis highlighted a clear separation between samples originating from CB and from pan-DCs upon stimulation. 890 genes, enriched for cell-cycle genes, were found to cause the separation (**Supplementary Figures S4A, B**). Similar to observations reported by Anselmi et al. (31), we attributed these genes to an *in vitro* differentiation signature and extracted them from the hierarchical analysis which translated into a treatment-dependent clustering of the samples (**Figure 5A**).

To further characterize transcriptional responses upon TLR9 or TLR7 stimulation of CB- and primary pDCs, we performed a DGE analysis comparing TLR-stimulated samples to their untreated counterparts. TLR9 or TLR7 treatment elicited a significant gene modulation for both primary pDCs and unprimed and primed CB-pDCs (**Figures 5B, C**). The majority of IFN-ɑ subtypes were significantly up-regulated upon treatment for both CB- and primary pDCs, in particular, *IFNA4* and *IFNA21* (**Supplementary Figure S4C**). Further, genes associated with pDC activation including the IFN responsive genes *IFIH1*, *STAT1*, *STAT2*, *MX1*, and *OAS1* and the inflammatory genes *NFкB*, *TRAF1*, and *ITGB8* were among the top up-regulated genes across all samples (**Figures 5B, C**; **Supplementary Figure S4C** and **Supplementary Table S3**). In addition, TLR7 treatment induced a stronger down-regulation of genes associated with cell cycle and metabolism compared to TLR9 treatment (**Figures 5B**, **C**; **Supplementary Figure S4D** and **Supplementary Table S3**).

Next, the expression of 34 genes associated with pDC activation and function were analyzed in detail across different conditions and samples (**Figure 5D**). The 8 selected IFN-α subtypes were induced upon TLR9 or TLR7 stimulation in CB-pDCs and primary pDCs, with a stronger expression observed in primed CB-pDCs already after 4 hrs. Primed CB-pDCs expressed 3 of the 4 *IFN-λ* subtypes in a stronger fashion upon TLR7 stimulation compared to unprimed CB-pDCs and primary pDCs. Conversely, primary pDCs strongly expressed *CCL3*, *CCL4*, and *CXCL10* compared to primed CB-pDCs, whereas TLR7-treated CB-pDCs were especially enriched in *CCL5* transcripts. *TNF-ɑ* transcripts could be detected across all conditions with an enhanced induction upon TLR9 treatment in contrast to our protein data showing a lack of TNF-ɑ secretion for CB-pDCs (**Figures 4C**, **5D**). TLR stimulation, especially TLR7 treatment, elicited the up-regulation of various genes encoding co-stimulatory molecules like *CD80*, *CD40*, and *CCR7* in CB-pDCs and primary pDCs. Corroborating the protein data shown in **Figure 4**, no differences in modulation of these genes were observed between unprimed or primed CB-pDCs. *GZMB* transcripts were up-regulated across samples with a stronger expression observed in primary pDCs. Finally, *ID2*, the *TCF4* repressor previously associated with pDC function, appeared highly up-regulated in TLR7-treated CB- and primary pDCs (**Figure 5D**).

Altogether these findings demonstrate a strong functional homology between CB-pDCs and primary pDCs upon TLR9 and TLR7 stimulation at the transcriptional level and confirm that IFN priming potentiates CB-pDC responses in a type I IFN specific manner.

Next, we performed a high throughput GSEA analysis with the BubbleGUM tool to determine pathways enriched in TLR-stimulated pDCs comparing them to their respective baseline condition, untreated unprimed CB-pDCs, and untreated primary pDCs (**Figure 5E**). Pathways associated with TLR stimulation and signaling and pDC inflammatory responses were strongly induced across all conditions including IFN-ɑ response, IRF7, STAT, and cytokine signaling pathways (4, 54, 55). TLR7-treated CB-pDCs were specifically enriched for the TNFR2, IL-6 STAT3, and IL-2 STAT5 pathways, suggesting a differential response to TLR stimulation in CB-pDCs compared to primary pDCs. Pathway analysis also revealed a potent modulation of several metabolic pathways as oxidative phosphorylation (OXPHOS) and mitochondrial respiration were strongly downregulated in primed TLR7-treated CB-pDCs in contrast to other conditions tested. Primed, TLR7-treated and TLR9-treated CB-pDCs and TLR9-treated primary pDCs displayed a repression of myelocytomatosis oncogene (Myc) target pathways (**Figure 5E**).

These results hint to a role of metabolic reprogramming and Myc pathways in the priming effect observed upon TLR7 treatment. Therefore, we conducted a DGE analysis comparing unprimed and primed conditions for the different stimulation types in CB-pDCs highlighting that untreated samples already showed the highest magnitude of up-regulated genes upon priming compared to TLR9 or TLR7 treatment (**Supplementary Figure S4E**). BubbleGUM analysis revealed that already in untreated CB-pDCs, IFN priming triggered pathways associated with an activation phenotype such as OAS, IFN-ɑ, and TNF-ɑ pathways. Interestingly, primed CB-pDCs across all TLR-treated conditions exhibited a robust down-regulation of Myc pathways confirming a potential role of Myc in the IFN priming effect. Similarly, these cells also repressed OXPHOS and associated mitochondrial respiratory pathways compared to their unprimed counterparts (**Supplementary Figure S4F**).

In conclusion, our transcriptional data confirm the strong resemblance between CB-pDCs and primary pDCs upon TLR stimulation and suggest a potential role of Myc pathways and metabolic reprogramming in the IFN priming effect.

### 3.6 CB-pDCs induce inflammation in co-culture with CRC tumor digests

**Figure 6:**
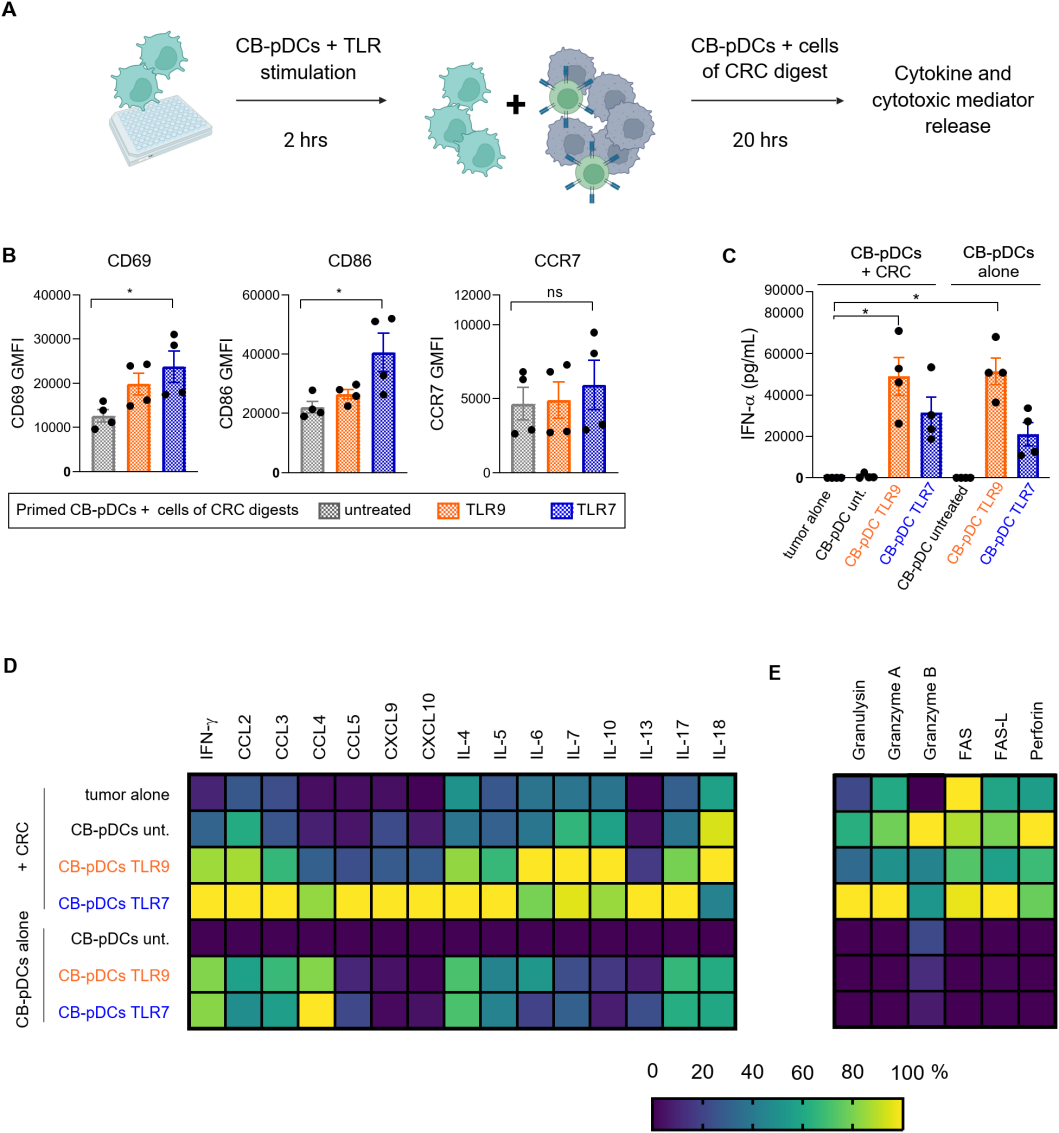
CB-pDCs can induce inflammation in co-culture with cells from CRC tumor digests. **(A)** Graphical representation of the experimental set-up of the CB-pDC/CRC co-culture. **(B)** Quantification of activation markers on CB-pDCs upon co-culture with CRC digests using flow cytometry. **(C)** Quantification of IFN-ɑ release in supernatant of CB-pDCs either from co-culture with CRC digests or cultured alone showing all indicated treatment groups. **(D, E)** Quantification of **(D)** cytokine and chemokine release and **(E)** release of cytotoxic mediators in supernatant of CB-pDCs either from co-culture with CRC digests or cultured alone of all indicated treatment groups. Colors displaying the minimum (0%) to maximum (100%) mean cytokine concentration per column. Results are shown as mean ± SEM of 2 independent experiments, with one independent CB donor and 4 independent CRC donors. *p>0.1, ns: not significant, one-way ANOVA with Tukey’s *post-hoc* test. See also **Supplementary Figure S5** and **Supplementary Table S1**.

We demonstrated that CB-pDCs can be differentiated in high numbers from human HSCs. Moreover, we extensively showed their resemblance to primary pDCs suggesting they are faithful surrogates. We next sought to test our initial hypothesis that CB-pDCs could be used as cell therapy to inflame the tumor microenvironment and activate effector cells such as NK cells. To achieve this, CB-pDCs were differentiated, sorted, primed and stimulated for 2 hrs before co-culture with cell suspensions from tumor digests from 4 CRC patients for 20 hrs (**Figure 6A** and **Supplementary Table S1**). As shown previously, TA-pDCs exhibit an unresponsive phenotype towards TLR stimulation, therefore we first examined the activation of CB-pDCs following 20 hrs co-culture with the cells derived from tumor digests. CB-pDCs were tested for the expression of the activation markers CD69, CD86, and CCR7 using flow cytometry (**Figure 6B**). After 20 hrs of co-culture, CB-pDCs displayed expression of these markers which was significantly enhanced for TLR7-treated CB-pDCs except for CCR7. As the main immunosuppressive characteristics of TA-pDCs is the loss of IFN-ɑ production upon TLR stimulation (**Figure 1C**), we evaluated IFN-ɑ secretion in co-culture and compared it to CB-pDCs stimulated under the same conditions (**Figure 6C**). In this case, stimulated CB-pDCs co-cultured with tumor cells successfully secreted IFN-ɑ in a comparable range to CB-pDCs alone, suggesting a sustained functional phenotype. To further confirm the pro-inflammatory features, we quantified cytokine and chemokine levels in the supernatant of the co-culture and compared it to CB-pDCs alone (**Figure 6D** and **Supplementary Figure S5A**). Interestingly, TLR7-treated CB-pDCs in co-cultures with the CRC tumor digest induced a broad range of cytokines and chemokines including the cytotoxicity-associated cytokine IFN-ɣ, as well as CCL5, CXCL9, and CXCL10, chemokines associated with T and NK cell recruitment and the pro-inflammatory cytokines IL-4, IL-5, and IL-17 (56–58). This effect was specific for TLR7, as TLR9-treated CB-pDCs in co-culture induced mainly cytokines such as IL-6, IL-10, and IL-18 (59). The presence of factors associated with effector cell functionality prompted us to additionally investigate the secretion of cytotoxic factors and mediators in the co-culture system (**Figure 6E** and **Supplementary Figure S5B**). Co-cultures with TLR7-treated CB-pDCs showed a trend towards enrichment of the cytotoxic mediators granzyme A, granulysin, and soluble FAS whereas untreated CB-pDCs in the co-cultures induced stronger granzyme B and perforin secretion.

Overall, these results illustrate that in co-culture with cells from tumor digests, pre-activated CB-pDCs maintain their inflammatory features and more importantly their ability to secrete type I IFN. Additionally, we demonstrate that TLR7-treated CB-pDCs promote a strong inflammatory environment in co-culture with cells from tumor digests of CRC patients suggesting a potential to recruit and activate effector cells.

## 4 Discussion

Due to their ability to secrete type I IFN and cross-talk with a broad range of immune cells, pDCs have been under scrutiny as targets for cancer immunotherapy (18, 60). Yet, we and others report that *in situ* activation of TA-pDCs remains challenging due to low numbers and an acquired immunosuppressive phenotype defined by impaired IFN-ɑ release (10, 12).

We hypothesized that vaccination with sufficient numbers of activated pDCs can promote an anti-tumor response by eliciting inflammation of the TME. Approaches based on primary pDCs and monocyte-derived DCs (mo-DCs) were previously described in melanoma and prostate cancer patients, however with the caveats of limited numbers of cells and poor homology to their blood counterparts, respectively (24, 61–65).

Here, we report an *in vitro* differentiation system supporting the differentiation of large numbers of functional CB-pDCs from HSCs. Moreover, we demonstrated extensively the resemblance of CB-pDCs at the phenotypic, transcriptional, and functional levels to primary pDCs, thus emerging as surrogates to investigate pDC pathophysiology and as potential cancer immunotherapy application.

Our *in vitro* differentiation protocol resulted in one of the highest pDC yields compared to literature data and significantly more than the 1-1.5x10^6^ primary pDCs that can be isolated from 200 mL of blood (28, 31–34, 66). Here, we validated a 2-step protocol consisting of an expansion and differentiation phase, the latter supplemented with FLT3-L, SCF, IL-7, ascorbic acid, GM-CSF, and SR-1. Although many protocols reported a role of GM-CSF and SR-1 in supporting *in vitro* pDC differentiation, we demonstrate here a paradoxical role of both factors in pDC differentiation and function (26, 30, 33, 67). In line with our results, Ghirelli and colleagues showed that GM-CSF enhanced pDC function (68, 69). The interplay of STAT3/STAT5 signaling is tightly regulated by FLT3-L and GM-CSF, respectively, and was shown to fine tune the balance between the pDC master transcription factor TCF4 and its antagonist ID2 (70–72). We hypothesize that GM-CSF supplementation elicits STAT5 signaling which in turn might increase ID2 expression and disrupt CB-pDC differentiation.

Our results suggest a similar effect of SR-1 contrasting with reports demonstrating its role in pDC differentiation but highlighting its potential to enhance CB-pDC function consistent with reports from Díaz-Rodríguez and colleagues (27). Recently, aryl hydrocarbon receptor inhibitors were shown to promote mo-DC function by repressing their tolerogenic functions at the epigenetic level (73). Therefore, SR-1 might act similarly during pDC differentiation. Our findings indicate that GM-CSF and SR-1 are not required for pDC differentiation but exert a beneficial influence on CB-pDC function.

Phenotypical and transcriptional comparison unequivocally confirmed the strong homology between CB-pDCs and primary pDCs. CB-pDCs recapitulated the expression of most pDC markers and genes including the master transcription factor TCF4 (74). CD304 and ILT7 being more prominently expressed in CB-pDCs and the latter being involved in pDC response upon infection, it would be interesting to investigate how this influences CB-pDC function (75). Moreover, GSEA analysis confirmed CB-pDC resemblance to their blood counterparts by comparing transcriptional signatures with the reference DC dataset by Villani et al. (43). Additionally, transcriptional analysis highlighted the concomitant differentiation of all conventional DC subtypes and the precursor population pre/AS-DCs in this system. This observation corroborated our phenotypical analysis and confirmed the strong myeloid component of this *in vitro* differentiation system consistent with other reports.

Our experiments also confirmed that CB-pDCs are equipped with functional TLRs enabling type I IFN, cytokine and chemokine responses as well as up-regulation of co-stimulatory molecules including CCR7. CB-pDCs responded to TLR stimulation similarly as primary pDCs, albeit without secretion of IL-6 and TNF-α while showcasing superior CCL4 and CCL5 production. Further, CB-pDCs were able to elicit hallmarks of NK cell activation, a key mechanism by which pDCs support antiviral immune responses (76, 77).

*In vitro* differentiated CB-pDCs depend on IFN priming to release IFN-ɑ, a concept primarily introduced by Laustsen and colleagues (33). In their study, primed *in vitro* differentiated pDCs showed enhanced expression of canonical pDC markers and co-stimulatory molecules as well as type I IFN secretion. Conversely, IFN priming solely unleashed IFN-ɑ secretion without further enhancing secretion of additional cytokines or co-stimulatory molecules in our hands. The phenotype of unprimed CB-pDCs however resembles TA-pDCs due to the lack of IFN-ɑ secretion. In line with this, He and colleagues reported similar observations with TA-pDCs in co-culture with patient tumor explants (78).

The evaluation of functional responses at the transcriptional level revealed that both unprimed and primed CB-pDCs responded to TLR stimulation by up-regulating characteristic gene sets and pathways associated with type I IFN and inflammatory responses including most IFN-ɑ subtypes as well as NFkB and STAT3/5 pathways. Additionally, primary pDCs were recently shown to upregulate ID2 upon CD40L stimulation, a feature also shared by CB-pDCs upon TLR7 activation (54). Moreover, in line with our observations at the protein level, various co-stimulatory molecules and chemokines were strongly up-regulated upon TLR stimulation independent of IFN priming. These results alongside the lack of modulation of *IRF7*, *TLR7*, or *TLR9* confirm the discrepancy from the IFN priming effect described by Laustsen and colleagues (33) which might be attributed to the differences between both culture systems.

GM-CSF supplementation was previously described to elicit strong up-regulation of *MYC (* 79). Additionally, Myc expression was reported to repress type I IFN responses (80, 81). In our hands, Myc-related pathways appeared consistently down-regulated in IFN-primed CB-pDCs. IFN-β and IFN-ɣ were both shown to inhibit Myc which might be an explanation for rescuing of type I IFN responses in CB-pDCs upon IFN priming (82, 83). However, in case Myc has the major influence on type I IFN induction, unprimed CB-pDC should not be able to secrete IFN-ɑ in our differentiation system. Moreover, IFN priming also represses pathways associated with mitochondrial metabolism, especially in combination with TLR stimulation. Interestingly, metabolic reprogramming was reported to be linked to substantial IFN-ɑ secretion by pDCs and exposure to type I IFN was previously shown to promote pDC activation via glycolytic switch (84–86). This indicates a potential involvement of both, Myc and metabolic reprogramming in the IFN priming effect observed here. Further work employing pharmacological targeting of Myc and mitochondrial respiration would be necessary to unravel the exact underlying mechanisms.

Taking advantage of large numbers of CB-pDCs and their robust resemblance to primary pDCs, CB-pDCs were tested as potential cellular cancer immunotherapy modality *ex vivo* in patient tumor explants. TLR7-treated CB-pDCs in co-cultures with CRC tumor digest induced a broad spectrum of cytokines and chemokines associated with T and NK cell trafficking and potential cytolytic function alongside macrophage and cDC recruitment (57, 58, 87–89). Contrasting with TA-pDCs, CB-pDCs maintained a robust pro-inflammatory phenotype in co-culture with CRC tumor digests suggesting that pre-activation limits the potential immunosuppressive effect of the tumor digests. However, it is important to consider that 2D cultures of tumor digests cannot recapitulate the entire complexity of the immunosuppressive TME (90, 91). The secretion profile specifically observed in the co-culture set-up might be attributed to bystander activation of immune cells present in the tumor digests like NK and T cells, confirming the ability of stimulated CB-pDCs to trigger inflammation in the TME. An alternative explanation could be that the TME exerted a differential influence on CB-pDCs, driving secretion of these factors. Nonetheless, factors such as IFN-ɣ, IL-7, granulysin, and granzyme A are uncharacteristic of pDCs and rather match effector cell profiles (3, 4, 92, 93). Overall our data support the hypothesis that CB-pDCs can promote inflammation and anti-tumor immunity. These results should be further substantiated in co-culture with other tumor indications and in 3D co-culture assays.

The source and scarcity of pDCs being the main obstacles to pDC vaccination, transfer of allogeneic CB-pDCs would represent a unique opportunity for cancer immunotherapy (23). Diaz-Rodriguez and colleagues reported no safety risks following systemic administration of TLR9-stimulated *in vitro* differentiated pDCs in NSG mice (27). Moreover, successful vaccination with allogeneic pDCs in melanoma patients with partially matched HLA-type established the feasibility of allogeneic pDC vaccines (94). Thus, we hypothesize that CB-pDCs might represent a potential therapeutic option with a promising safety profile. Finally, it is important to note that the use of murine stromal cells and FCS in this differentiation protocol as well as the requirement to sort CB-pDCs prior to use remain key limitations inevitable to overcome for a potential clinical application.

## Data Availability

This paper does not report original code. The raw and processed scRNAseq datasets can be found at https://doi.org/10.6084/m9.figshare.25561950.v1. Any additional information required to reanalyze the data reported in this paper is available upon request.
