# Supplementary material for: Human CD34+-derived plasmacytoid dendritic cells as surrogates for primary pDCs and potential cancer immunotherapy
Source: Front Immunol. 2024 Nov 7;15:1433119. doi: 10.3389/fimmu.2024.1433119 (PMC11578708; doi:10.3389/fimmu.2024.1433119)
Supplement: Supplementary file 1 [file DataSheet1.pdf]

Supplementary Material

Human CD34^{+}-derived plasmacytoid dendritic cells as surrogates for primary pDCs and potential cancer immunotherapy

Giovanna Fiore[1], Wolfgang Weckwarth[1], Kerstin Paetzold[1], Llucia Albertí Servera[2], Manuela Gies[1], Jakob Rosenhauer[1], Martina Antoniolli[1], Sina Nassiri[2], Stephan Schmeing[2], Steffen Dettling[1], Bhavesh Soni[3], Meher Majety[1], Anne B. Krug[4], Sabine Hoves[1] and Monika Julia Wolf[1,*]

*** Correspondence:** Monika Wolf: monika.wolf@roche.com

Supplementary Figure S1: Generation of *in vitro* differentiated CB-pDCs from CD34^{+} HSCs. (A) CB-derived CD34^{+} HSCs (5,000/well) were expanded for 7 days in expansion media. Graph represents fold change proliferation during the expansion phase of all cells on day 7. See also Supplementary Table S2 for calculation of absolute numbers of cells generated. (B) Dot plots show CB-pDC populations defined as CD123^{+}CD303^{+} (of Living^{+}CD45^{+}Lin^{-}) from one representative donor on day 19 comparing supplementation of SR-1 and GM-CSF separately or in combination. (C) Gating strategy for CB-pDCs. Dot plots representative of one donor illustrating the gating strategy for CB-DCs. CB-DCs were defined as Living^{+}CD45^{+}Lin^{-}, CB-cDC1 as CD141^{+}CLEC9A^{+}, CB-cDC2 as CD1c^{+}CD11c^{+} and CB-pDC as CD123^{+}CD303^{+}. (D) Histogram displaying CD11c expression in the mixed cells (left), on CB-cDC2 (blue) and CB-pDCs (orange) (right). (E) Bar plot displaying the frequency of CB-cDC1s, CB-cDC2s, and CB-pDCs as determined by flow cytometry (n=5). (F) Graph depicts frequencies of CD4, CD45RA, ILT-7, and CD304. Results are shown as mean ± SEM of 2 independent experiments, 8 independent CB donors, and 3 independent pDC donors.

Supplementary Figure S2: CB-pDCs resemble primary pDCs at the transcriptional level. (A) Frequency of cDC1s, cDC2s, and pDCs from pan-DCs enriched from PBMCs as determined by flow cytometry (n=3). (B) scRNA sequencing was performed on *in vitro* differentiated CB cells and primary pan-DCs. UMAP shows the distribution of cells from CB-pDC and pan-DC samples. (C) Stacked bar plots depicting the percentage of the immune cell subsets for each CB and pan-DC donor. Results are shown from 3 independent CB donors (D13, D21, and D26) and 3 independent pan-DC donors (D1, D3, and D4). (D) UMAPs of CB cells and pan-DCs showing the expression values of described pDC genes in Log(CP10k+1). (E) Dot plot illustrating the fraction of positive cells and their mean expression of described pDC genes for CB-pDCs (D13, D21, and D26) and primary pDCs (D1, D3, and D4). Dot size indicates fraction of positive cells, color indicates mean expression levels. Results of 3 independent untreated, unprimed CB donors and 3 independent pan-DC donors are depicted.

Supplementary Figure S3: CB-pDCs show key functional features resembling primary pDCs. (A) Sorting strategy for CB-pDCs. Upper dot plots are representative of one donor illustrating the strategy for isolation of CB-pDCs, which were identified as CD45^{+}Lin^{-}CD123^{+}CD45RA^{+}AXL^{-}CD327^{-}. Lower dot plots show matching fluorescence minus one control (FMO) for CB-pDC sorting strategy. Graph (right) represents the frequency of CD45^{+}Lin^{-} and CB-pDCs from the morphology

“cell” gate. (B) Dot plot of CB-pDC analysis after sorting of one representative donor. (C) Titration of IFN for priming. CB-pDCs were sorted and primed with 1 μg/mL, 100 ng/mL, 10 ng/mL, or 1 ng/mL of IFN-β and IFN-γ for 72 hrs. Subsequently, primed cells were activated with TLR9 or TLR7 agonists for 24 hrs. Upper graphs show the quantification of IFN-α release in supernatant upon TLR9 (left) or TLR7 (right) stimulation. Lower graphs show the quantification of CD40 (left) and CD80 (right) on the surface of pDCs upon TLR9 or TLR7 stimulation. Gray boxes indicate IFN priming conditions. Results are shown as mean ± SEM of 1 or 2 independent experiments with 6 (IFN-α) or 3 (surface marker) independent CB donors. (D) Unprimed and primed CB-pDCs as well as primary pDCs were activated with TLR9 or TLR7 agonists for 24 hrs. Quantification of CCL4 (left), IL-6 (middle) and IL-12p70 (right) in supernatant. Results are shown as mean ± SEM of 2 independent experiments, 6 independent CB donors and 4 independent pDC donors. $*p>0.1$, $**p>0.01$, two-way ANOVA with Tukey’s *post-hoc* test.

Supplementary Figure S4: TLR stimulation activates similar genes and pathways in CB-pDCs and primary pDCs. Unprimed and primed CB-pDCs and primary pDCs were activated with TLR9 or TLR7 agonists for 4 hrs and scRNA sequencing was performed. (A) Cluster dendrogram representing the hierarchical clustering of CB-pDC and primary pDC samples upon treatment before extracting the *in vitro* differentiation culture signature composed of 890 genes. (B) Top 10 associated pathways (Gene Ontology) for the *in vitro* differentiation signature. (C, D) Heatmap represents (C) the up-regulation and (D) down-regulation of the top 60 genes for unprimed and primed CB-pDCs and primary pDCs upon TLR9 or TLR7 treatment relative to control condition (untreated unprimed for CB-pDCs and untreated for primary pDCs). List of genes is shown in Supplementary Table S3. (A-D) Results of 3 independent CB donors and 3 independent pan-DC donors are shown. (E) Volcano plots displaying differentially expressed genes comparing unprimed and primed conditions to untreated (upper left), TLR9 (lower left) or TLR7 (right) stimulation for CB-pDCs. Colors display significance (orange) and significance and high log fold change (red; FDR <+ 5%, abs(log2FC) >= 1), n.s. not significant (gray). (F) BubbleMap showing the most relevant pathways enriched in primed untreated, TLR9- and TLR7-activated CB-pDCs compared to unprimed matching conditions. Color code as explained within the figure, increasing intensity represents stronger enrichment in treated groups (red) or in controls (blue) with bubble sizes corresponding to the absolute values for the normalized enrichment score. (E-F) Results of 3 independent CB donors are shown, with unprimed and primed CB cells with or without activation with TLR9 or TLR7 agonists for 4 hrs.

Supplementary Figure S5: CB-pDCs can induce inflammation in co-culture with CRC tumor digests. Sorted and primed CB-pDCs were activated with TLR7 or TLR9 agonists for 2 hrs, then co cultured for 20 hrs with single cell suspensions derived from CRC tumor digests. (A, B) Quantification of (A) cytokine and chemokine release and (B) release of cytotoxic mediators in supernatant of CB-pDCs either from co-culture with CRC tumor digests or cultured alone of all indicated treatment groups. Results are shown as mean ± SEM of 4 independent experiments, with one independent CB donor and 4 independent CRC tumor digest samples. $*p<0.05$, $**p<0.01$, $***p<0.001$, $****p<0.0001$, one-way ANOVA with Tukey’s *post-hoc* test or Kruskal-Wallis test with Dunn’s *post-hoc* test.

Supplementary Figure S1

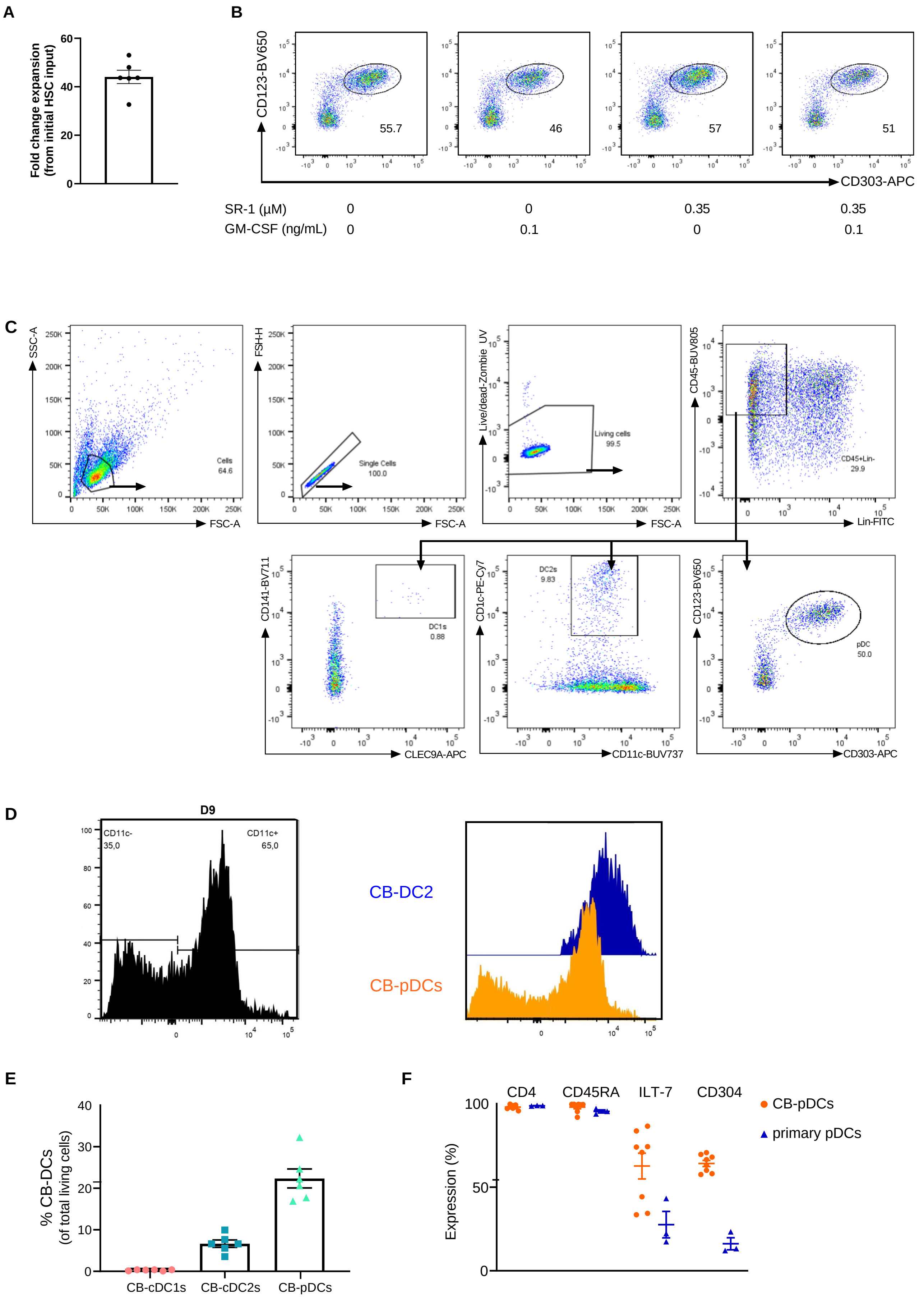

Supplementary Figure S2

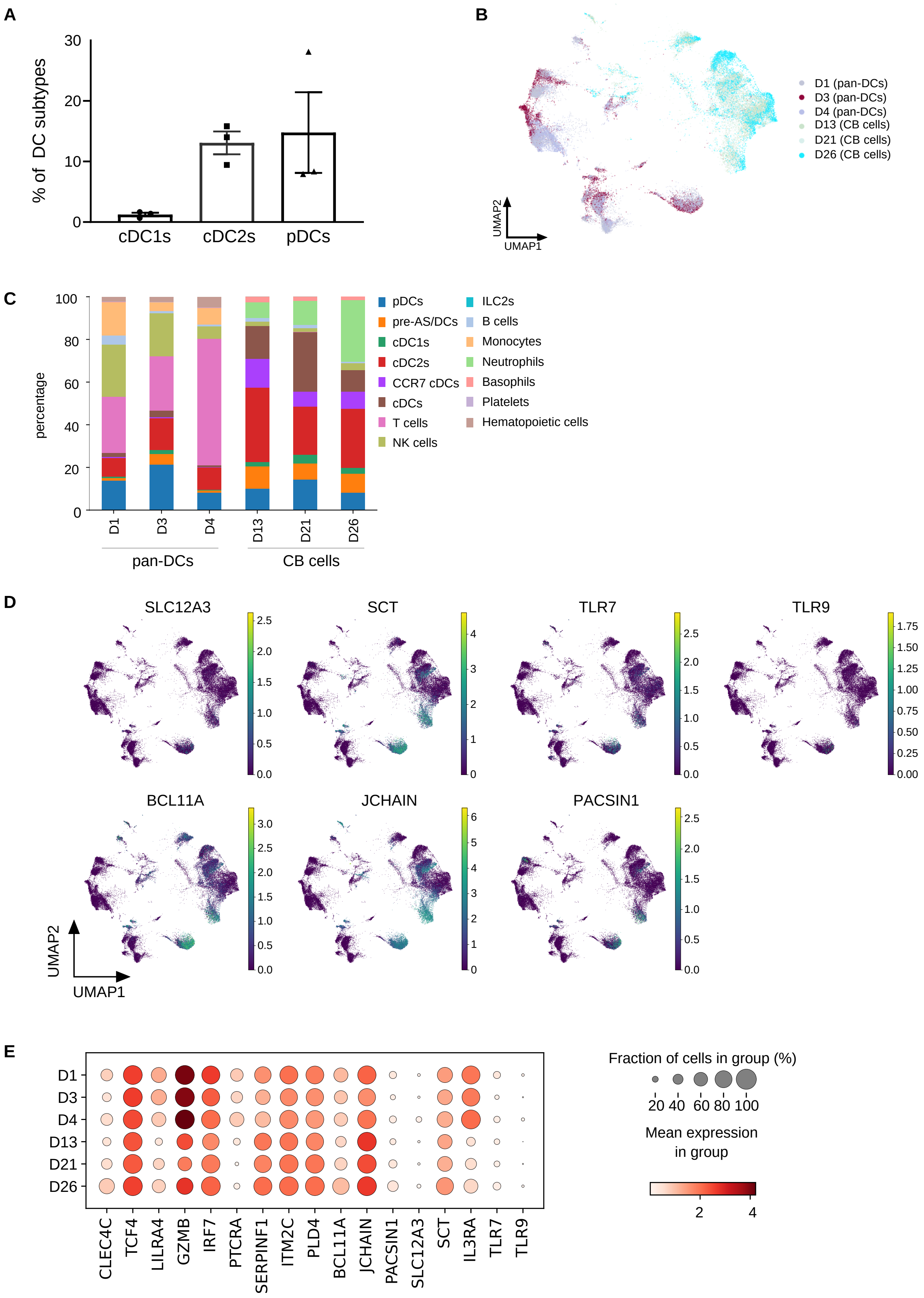

Supplementary Figure S3

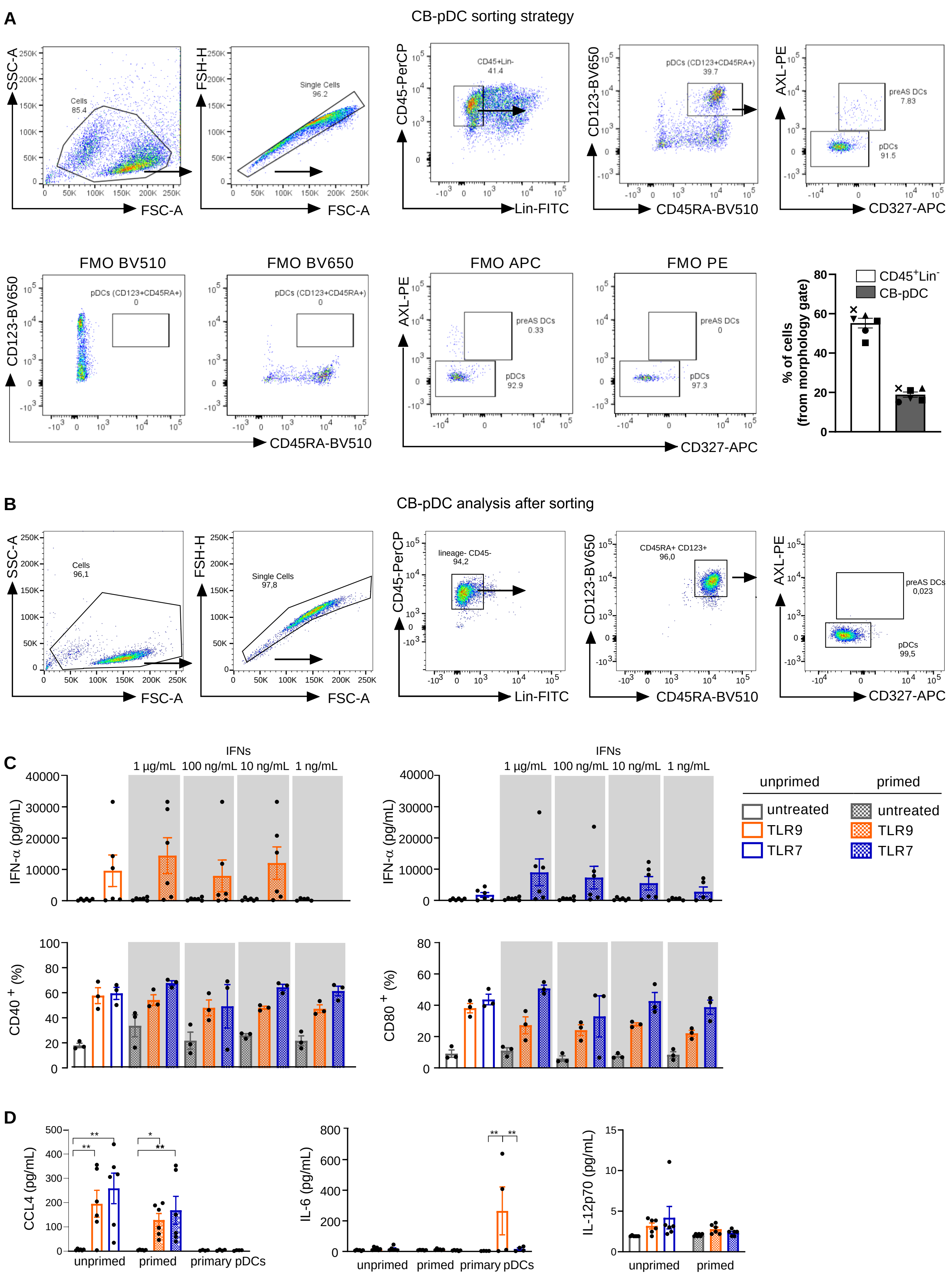

Supplementary Figure S4

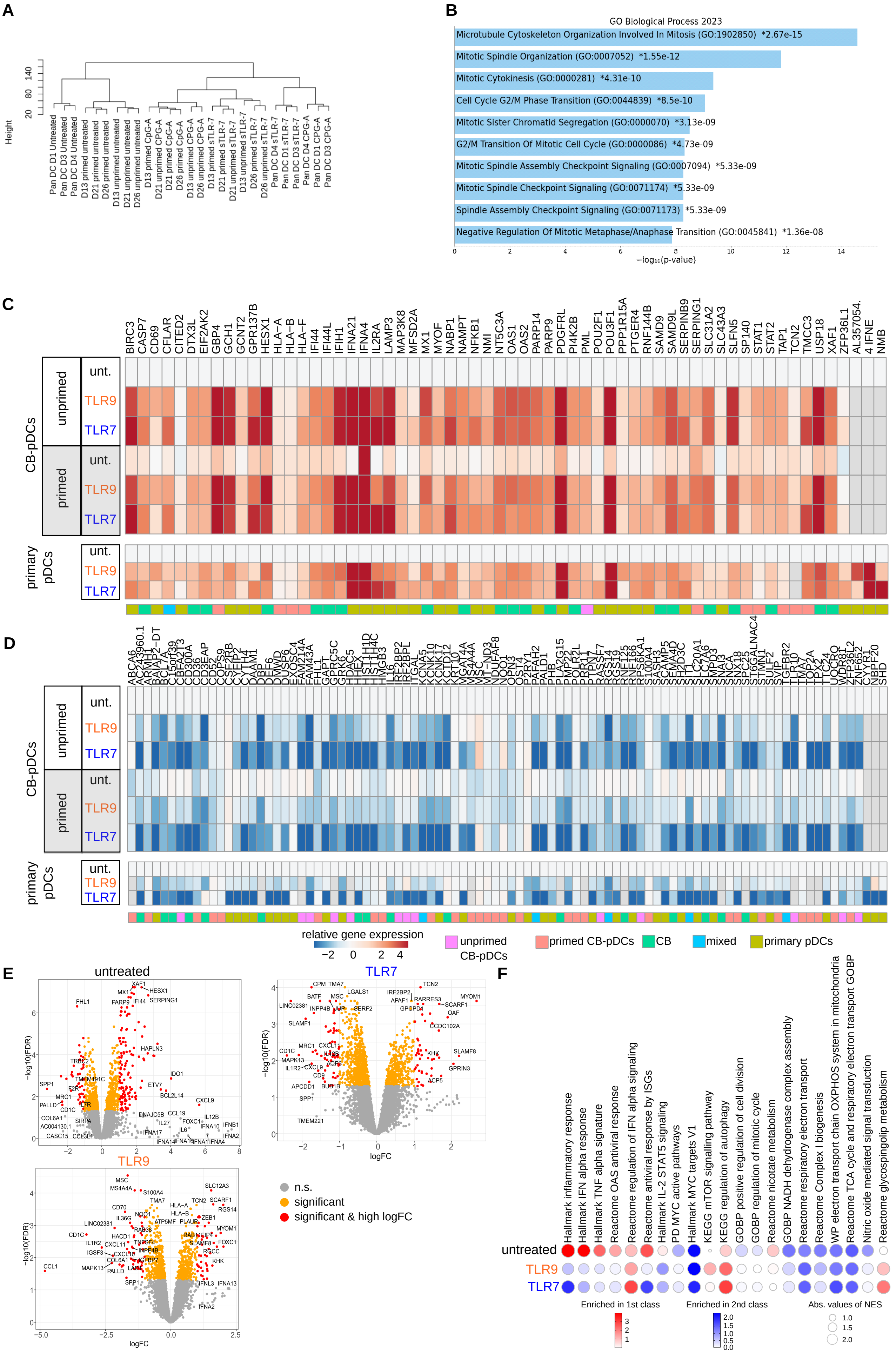

Supplementary Figure S5

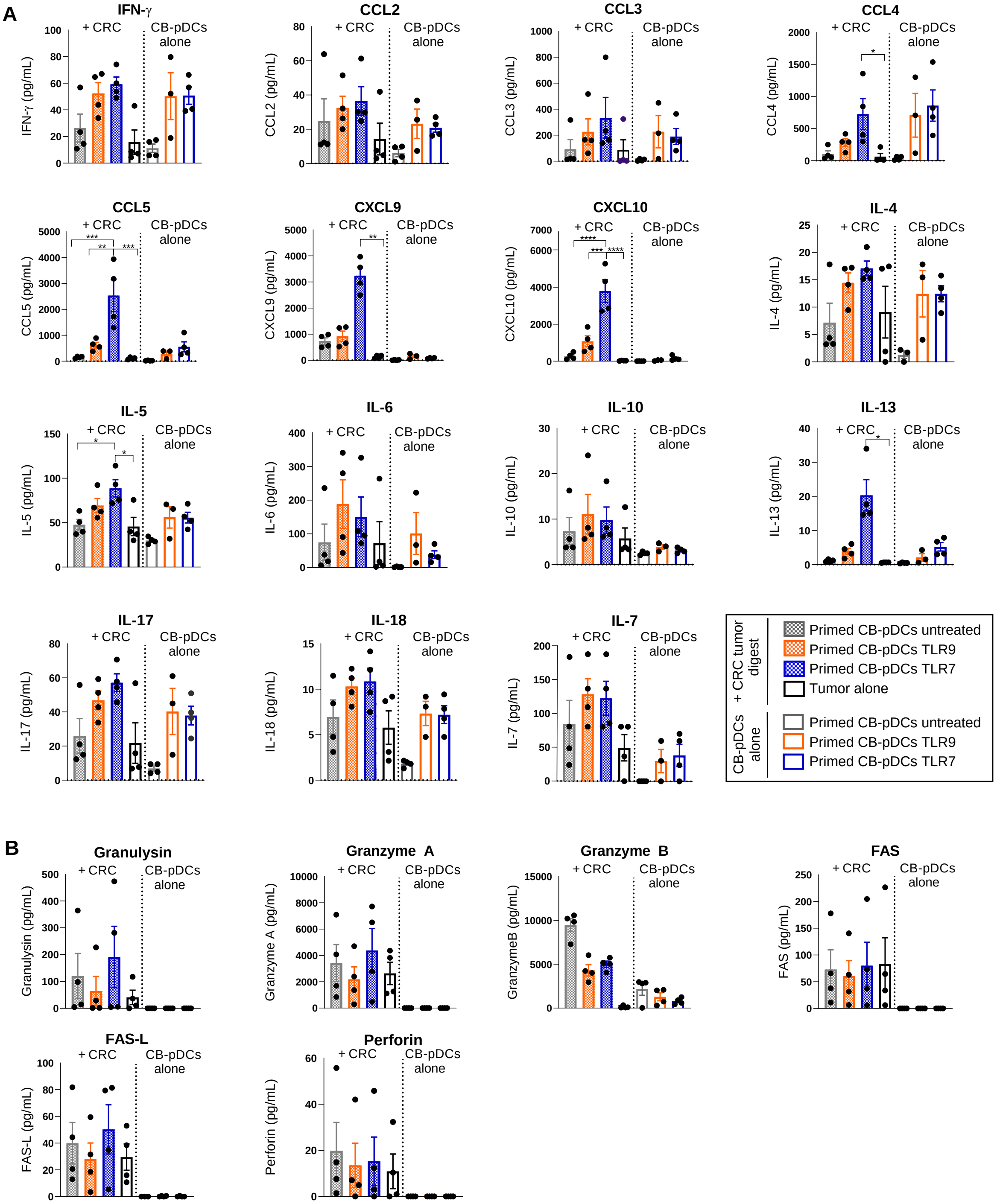

Supplementary Table S1: Overview of tumor samples used in this manuscript

Sample ID	Tumor indication	Gender	Age	Experiment
O1	Ovarian cancer, carcinosarcoma (MMMT)	Female	77	Flow cytometry screening (Fig.1)
O2	Ovarian cancer, serous carcinoma	Female	53	Flow cytometry screening (Fig.1)
O3	Ovarian cancer, cystadenocarcinoma	Female	66	Flow cytometry screening (Fig.1)
A7083	Colorectal cancer, adenocarcinoma - partially mucinous	Female	82	Co-culture with CB-pDCs (Fig.6)
Z1846	Colorectal cancer, adenocarcinoma	Male	56	Co-culture with CB-pDCs (Fig.6)
Z1854	Colorectal cancer, adenocarcinoma	Female	76	Co-culture with CB-pDCs (Fig.6)
B2915	Colorectal cancer, adenocarcinoma	Male	55	Co-culture with CB-pDCs (Fig.6)
C1	Colorectal cancer, adenocarcinoma	Male	58	Flow cytometry screening (Fig.1)
C2	Colorectal cancer, adenocarcinoma	Female	76	Flow cytometry screening (Fig.1)
C3	Colorectal cancer, adenocarcinoma	Female	65	Flow cytometry screening (Fig.1)
C4	Colorectal cancer, adenocarcinoma	Female	50	Flow cytometry screening (Fig.1)
B1	Breast cancer	Female	82	Flow cytometry screening (Fig.1)
B2	Breast cancer	Female	67	Flow cytometry screening (Fig.1)
B3	Breast cancer	Female	59	Flow cytometry screening (Fig.1)
B4	Breast cancer	Female	37	Flow cytometry screening (Fig.1)
22473-02	Breast cancer	Female	unknown	Flow cytometry screening and TLR7 stimulation (Fig.1)
22473-03	Breast cancer	Female	unknown	Flow cytometry screening and TLR7 stimulation (Fig.1)
22473-05	Breast cancer	Female	unknown	Flow cytometry screening (Fig.1)
22473-06	Breast cancer	Female	unknown	Flow cytometry screening and TLR7 stimulation (Fig.1)
22473-07	Breast cancer	Female	unknown	Flow cytometry screening (Fig.1)
H1	Head and neck cancer	Female	61	Flow cytometry screening (Fig.1)
H2	Head and neck cancer	Male	71	Flow cytometry screening (Fig.1)
H3	Head and neck cancer	Male	65	Flow cytometry screening (Fig.1)
H4	Head and neck cancer	Male	82	Flow cytometry screening (Fig.1)
L1	Lung cancer	Male	62	Flow cytometry screening (Fig.1)
L2	Lung cancer	Female	60	Flow cytometry screening (Fig.1)
L3	Lung cancer	Female	61	Flow cytometry screening (Fig.1)
L4	Lung cancer	Male	48	Flow cytometry screening (Fig.1)

Supplementary Table S2: Example of calculations to determine the absolute numbers of cells generated with the *in vitro* differentiation protocol

Donor ID	Expansion rate (fold change) during the Expansion phase[1]	Proliferation rate (fold change) during the Differentiation phase[2]	% of CB-pDCs (of total living cells)[3]	Count of CB-pDCs for HSC input	Count of CB-pDCs for 10,000 HCS input
D30	30	17.04	20.06	103	$1.03x10^6$
D12	43.46	38.75	17.8	300	$3x10^6$
D36	48.27	35.84	16.9	292	$2.9x10^6$
D21	28	19.76	24.6	136	$1.3x10^6$

[1] Expansion rate in fold change: defined as number of cells harvested at day 7 after the expansion/number of cells plated at day 0

[2] Proliferation rate in fold change: defined as number of cells harvested at day 19 after the differentiation/number of cells plated at the begining of the differentiation (day 7)

[3] % of CB-pDCs (of total living cells) with CB-pDCs defined as $CD45^+Lin^-CD303^+CD123^+$ by flow cytometry

Calculation example for Donor 30:

1 HSC x 30 fold expansion rate = 30 HSCs, 30 HSCs x 17.04 fold proliferation rate = 511.2 cells. Of these cells, 20.06% are CB-pDCs, meaning 102.5 are CB-pDCs.

For Donor 30, 1 HSC input at the begining of the experiment yield in a total of 102.5 CB-pDCs

Supplementary Table S3: Top 25 differentially expressed genes in primary pDCs and CB-pDCs upon TLR stimulation

	Primary pDCs TLR9		Primary pDCs TLR7		CB-pDCs unprimed TLR9		CB-pDCs unprimed TLR7		CB-pDCs primed TLR9		CB-pDCs primed TLR7	
	gene	Log FC	gene	Log FC	gene	Log FC	gene	Log FC	gene	Log FC	gene	Log FC
**Top 25 upregulated genes**	IFNA2	18.6	IFNA2	12.7	IFNA2	15.6	IFNA10	14.5	IFNA2	17.2	IFNA10	16.4
	IFNA10	17.2	IFNA10	12.2	IFNA10	15	IL12B	13.8	IFNA10	16.7	IFNA8	15.7
	IFNA8	17.2	IFNA8	11.5	IFNA8	14.9	CCL1	13.8	IFNA8	16.6	IFNA2	15.5
	IFNA17	16.3	IFNA5	10.9	IFNA4	14.2	IFNA8	13.7	IFNA4	16.2	IFNA1	14.5
	IFNB1	15.8	IFNA1	10.7	IFNA17	14	IFNA2	13.6	IFNA17	15.8	IFNA4	14.4
	IFNA5	15.8	IFNA4	9.5	IFNA5	13.8	IFNL3	12.7	IFNA5	15.5	IFNA17	14
	IFNA4	15.7	IFNB1	9.5	IFNA1	13.3	IFNA4	12.4	IFNA1	15.3	IFNA5	14
	IFNA1	15.7	AC099342.1	9	IL12B	13	IFNA1	12.4	IFNA21	15	IFNL3	13.6
	IFNA21	15.4	POU3F1	8.8	IFNA21	13	IFNA5	12.2	IFNA16	15	IFNA16	13.3
	IFNA6	15.4	IFNA13	8.6	IFNW1	12.7	CXCL11	12.2	IFNW1	14	FOXC1	13.1
	IFNA7	15.3	IFNA14	8.6	IFNA16	12.7	IFNA17	12.1	IFNA7	13.9	IFNA21	13.1
	IFNA14	15.2	IFNA21	8.3	IFNA14	12.4	IL36G	11.8	IFNA14	13.6	IFNA13	12.7
	IFNA16	15.2	ITGB8	8.3	IFNA7	12.2	CXCL10	11.6	IFNL3	13.6	IL12B	12.4
	IFNW1	14.4	IFNA17	8.2	CCL1	12.2	FOXC1	11.6	IFNA13	13.5	IFNW1	12.3
	IFNA13	14.4	IFNA16	7.8	IFNL3	11.8	CXCL9	11.5	IL12B	12.5	CCL1	12.3
	IFNE	12.4	IFNW1	7.6	CXCL11	11.7	CCL5	11.4	IFNL1	12.1	IFNA7	12.2
	AC099342.1	8.9	CCL19	7.5	CXCL10	11.7	IL27	11.3	IFNB1	12	IFNA14	11.5
	POU3F1	8.1	IFNA7	7.4	IFNA13	11.3	IFNA16	11.2	FOXC1	12	IL27	11.5
	SLC1A2	7.1	SLC1A2	7.4	CXCL9	10.9	IFNA13	11.1	IL27	10.9	IFNL1	11.3
	AC092723.3	7	ENTPD2	7.3	IFNB1	10.6	IFNA21	10.9	CXCL10	10.1	CCL5	11.3
	CCL4L2	6.7	IL36G	7.1	IL27	10.5	IFNW1	10.9	CXCL11	10.1	IL36G	10.8
	CCL3L1	6.3	IL6	7	IL36G	10.3	IL6	10.8	IL6	10	IFNB1	10.6
	IL36G	6	SOX5	7	FOXC1	9.9	IFNA7	10.5	CXCL9	9.8	CXCL11	10.5
	IL6	6	PDGFRL	6.9	IL6	9.9	IFNA14	9.9	CCL5	9.4	IL6	10.2
	CCL4	5.9	ZFPM2	6.9	IFNL1	9.9	MIA	9.9	IL36G	8.7	CXCL9	10
**Top 25 downregulated genes**	RHOB	-1.6	PDGFB	-4.5	CEBPA	-1.9	CCR2	-4.3	RHOBTB2	-1.9	APCDD1	-4.4
	PMP22	-1.6	BAIAP2-DT	-4.5	AC090152.1	-1.9	KCNK17	-4.4	TMEM221	-1.9	GTSE1	-4.4
	NLRC3	-1.6	CSF1R	-4.6	GPRC5C	-1.9	CEACAM21	-4.4	CD276	-1.9	HIST1H3B	-4.4
	PLAU	-1.6	TGFBR2	-4.7	MAFB	-2	HRH2	-4.4	KCNK10	-2	KCNK17	-4.5
	LCK	-1.6	KIAA1211L	-4.8	RPP25	-2	CD300A	-4.5	APCDD1	-2	CIT	-4.5
	SCAMP5	-1.7	LTB	-4.8	CCR2	-2	PMP22	-4.5	FILIP1L	-2	CENPA	-4.6
	FADS1	-1.7	ST6GALNAC	-4.9	RHOBTB2	-2	DUSP7	-4.5	CD36	-2	DLGAP5	-4.7
	HRH2	-1.7	AC083949.1	-4.9	HRH2	-2	RGCC	-4.5	SIGLEC6	-2	CENPF	-4.7
	SIT1	-1.7	TCL1A	-5	PLA2G15	-2.1	SERPINF2	-4.6	CD300C	-2.1	PROC	-4.7
	P2RY1	-1.7	DBN1	-5.1	TLR10	-2.1	DAB2	-4.6	SIT1	-2.1	BUB1B	-4.8
	AC090152.1	-1.7	AC119428.2	-5.3	LBH	-2.1	KIF18B	-4.6	DBP	-2.1	MAFB	-4.9
	DBN1	-1.8	FAM43A	-5.6	RGCC	-2.1	BLK	-4.6	TMEM191B	-2.1	NLRC3	-4.9
	GRK6	-1.8	NCR2	-5.6	IL16	-2.1	FAM43A	-4.7	SMOX	-2.1	NEK2	-4.9
	TRAV22	-1.8	KCNK17	-6	BCL7A	-2.2	TMEM221	-4.8	TRBC2	-2.2	MS4A4E	-4.9
	PAFAH2	-1.9	KLHL33	-6.1	RAB11FIP4	-2.2	KCNA5	-4.9	MT1X	-2.2	TRAV22	-5
	LRRC25	-1.9	SPIB	-6.3	LRRC25	-2.3	TRAV22	-4.9	DAB2	-2.3	BLK	-5
	RNF44	-1.9	HBEGF	-6.3	DUSP7	-2.3	LBH	-4.9	CD3EAP	-2.3	DAB2	-5
	BBC3	-1.9	RNF165	-6.4	SIT1	-2.4	PROC	-5	MAFB	-2.4	ARHGEF39	-5.1
	FAM43A	-2	SIT1	-6.5	SNAI3	-2.4	BIK	-5.1	NLRC3	-2.4	KIF20A	-5.2
	BAIAP2-DT	-2	HRH2	-6.6	KCTD12	-2.4	RHOBTB2	-5.3	PMP22	-2.4	SNAI3	-5.2
	CD3EAP	-2	HCAR1	-6.9	NLRC3	-2.5	SNAI3	-5.5	MS4A4E	-2.5	SIT1	-5.3
	CYYR1	-2	TRAV22	-7	FAM43A	-2.5	NLRC3	-5.5	NT5DC2	-2.5	LRRC25	-5.5
	RGS14	-2.3	KCNA5	-7.1	DBP	-2.6	ARHGEF39	-5.6	F2R	-2.6	RHOBTB2	-5.6
	RPP25	-2.4	TTC24	-7.4	CD3EAP	-2.7	SIT1	-6	LRRC25	-2.7	KCNA5	-5.7
	THBS1	-2.5	DAB2	-7.5	RGS14	-2.9	LRRC25	-6.3	MT1E	-2.9	TMEM221	-6.4
